# Design, validation, and application of a 1K liquid chip for genome-wide association analysis in Alpine Merino sheep

**DOI:** 10.3389/fvets.2025.1690580

**Published:** 2025-12-18

**Authors:** Tingting Guo, Chao Yuan, Jianbin Liu, Bohui Yang

**Affiliations:** 1 Lanzhou Institute of Husbandry and Pharmaceutical Sciences, Chinese Academy of Agricultural Sciences, Lanzhou, China; 2 Sheep Breeding Engineering Technology Research Center of Chinese Academy of Agricultural Sciences, Lanzhou, China; 3 Key Laboratory of Animal Genetics and Breeding on Tibetan Plateau, Ministry of Agriculture and Rural Affairs, Lanzhou, China

**Keywords:** fine wool sheep, targeted sequencing, genotyping, liquid chip, genome-wide association analysis

## Abstract

Because wool is an important animal fiber and mutton is rich in nutrients, fine wool sheep are one of the most economically important livestock. China has been selecting and breeding high-quality fine wool sheep breeds since the 1950s and is the largest processing and consuming country for fine wool sheep. In this study, blood samples were collected from Alpine Merino sheep (AMS), Chinese Merino sheep (CMS), Aohan fine wool sheep (AHS), and Qinghai fine wool sheep (QHS). Genomic DNA was extracted and subjected to genome resequencing. GenoBait technology was used for probe design and site optimization, followed by synthesis of a low-density liquid-phase chip. Finally, 409 AMS were randomly selected to verify the chip. The results showed that a total of 1,012 single-nucleotide polymorphism sites (SNPs) were screened and retained for the synthesis of a low-density liquid chip for genome-wide selection and population genetics structure analysis. This chip can provide a useful tool for genome analysis of fine wool sheep and lay a solid foundation for subsequent breeding work.

## Introduction

Fine-wool sheep form the cornerstone of the natural fiber industry, with their fine wool serving as a vital raw material for high-end textiles. Given their economic value, wool yield and quality have become core breeding objectives for fine-wool sheep, prompting the development of specialized genetic evaluation frameworks for these traits. The introduction of genomic selection technology into fine-wool sheep breeding has significantly accelerated genetic progress in economic traits, encompassing disease resistance, growth performance, and wool characteristics. Genome-wide selection enhances the precision of traditional genetic evaluations by integrating genomic information, enabling more accurate parameter estimation. Since Illumina launched the first sheep chip (Ovine SNP 50 Genotyping BeadChip v1) in 2009, multiple commercial sheep chips have been successfully developed. With advances in high-throughput sequencing and data analysis technologies, liquid-phase breeding chips based on targeted capture sequencing (genotyping by target sequencing, GBTS) have experienced rapid development as a new technology in recent years (1). GBTS technology is a technique for deep resequencing of target sites. The technical system is completely based on independent research and development results, is not restricted by foreign patented technology, does not rely on foreign equipment, and has the advantages of flexible operation, accuracy, reliability, low cost, and a wide application range. GBTS offers multiple advantages, including flexible marker addition and deletion and highly efficient detection, and has been widely applied in genomic selection for livestock species such as chicken (2), pigs (3), cattle (4), and aquatic animals (5). Whole genome selection breeding (GS) is a more efficient and accurate breeding method that uses high-density markers covering the whole genome for selective breeding after traditional breeding and molecular marker-assisted breeding (MAS). It can shorten the generation interval through the implementation of early selection, improve genetic estimated breeding value (GEBV), accelerate the genetic progress, and truly realize genomic technology to guide breeding practices. Due to the high cost of resequencing and high-density solid-phase gene chip detection, it is difficult for researchers and breeding companies to perform large-scale genome-wide selection breeding. Consequently, the development of low-density liquid-phase chips based on GBTS has become an inevitable trend. This study developed and designed a 1K functional marker site set of fine wool sheep using single nucleotide polymorphisms (SNPs) related to important economic traits of fine wool sheep and applied it to genome-wide association analysis (GWAS), which not only verifies the selected functional marker sites but also provides a technical means for functional markers, candidate gene screening, and genomic selection of fine wool sheep.

## Materials and methods

### Sample collection

All experimental protocols and procedures were approved by the Institutional Animal Care and Use Committee of the Lanzhou Institute of Husbandry and Pharmaceutical Science of the Chinese Academy of Agricultural Sciences (approval no. NKMYD201805; approval date: October 18, 2018). Whole-genome resequencing data from the chip design section were collected from 460 individuals in four fine wool sheep populations, including 220 Alpine Merino sheep (AMS; 75 males and 145 females; Huangcheng, Gansu, China), 120 Chinese Merino sheep (CMS; 60 males and 60 females; Gongnaisi, Xinjiang, China), 60 Aohan fine wool sheep (AHS; 30 males and 30 females; Chifeng, Inner Mongolia, China), and 60 Qinghai fine wool sheep (QHS; 30 males and 30 females; Sanjiaocheng, Gangcha, China). All the sheep were randomly selected without pedigree information. Blood samples for microarray validation were obtained from 409 additional AMS females from Huangcheng, Gansu, China. Blood and wool samples were collected from all individuals.

### Phenotypic data acquisition

Birth, weaning (3.5 months), yearling (12 months), and adult (30 months) weights of all sheep were measured using an electronic scale, and sex was recorded. Birth weights were recorded within 0.5 h of birth, and weaning, yearling, and adult weights were measured after 12 h of fasting. Wool samples were subjected to objective testing at the Quality Supervision, Inspection, and Testing Center for Animal Fur and Products of the Ministry of Agriculture (Lanzhou, China). The testing of wool traits was carried out according to relevant China national standards: Staple length (SL) (GB/T 6976-2007), clean fleece weight rate (CFWR) (GB/T 6978-2007), staple strength (SS) (GB/T 13835.5-2009), fleece extension rate (FER) (GB/T 13835.5-2009), fiber diameter (FD) (GB/T 10685-2007), and coefficient of variation of fiber diameter (FD-CV) (GB/T 10685-2007). Phenotypic data on body weight, shearing volume, and horn type were collected from the fine wool sheep production performance measurement field and shearing site. All data obtained from the tests and measurements were analyzed using SPSS software (version 20.0; SPSS Inc., Chicago, IL, USA) for basic statistics, including maximum value (Max), minimum value (Min), mean value (Mean), and coefficient of variation (CV). All phenotypic data were tested for normal distribution and outliers were excluded, and then analyzed and plotted by Pearson correlation using the cor function in R (version 4.0.3) (6, 7).

### Selection of candidate SNPs from whole genome resequencing

Blood samples were subjected to 5 × resequencing using a HiSeq X Ten (Illumina, San Diego, CA, USA). The detailed sequencing information can be found in our previous study (8, 9). High-quality sequencing data were aligned to the reference sheep genome assembly Oar_v4.0 (GCF_000298735.2) using the Burrows-Wheeler Aligner (BWA) software (v0.7.11) (10) (parameter: mem-t 4-K 32-M). Duplicates were removed using SAMtools (10) (parameter: rmdup). Finally, the sample alignment rate were analyzed statistically. We used SAMtools (11) to detect SNPs in the population samples and obtain high-quality SNPs through the following filtering and screening approach: (i) the support number (coverage depth) of SNP was >3; (ii) the proportion of MIS (missing) was < 10%; (iii) minimum allele frequency (MAF) was >5%. ANNOVAR is an efficient software tool for functionally annotating genetic variants detected across multiple genomes (12). Therefore, we used the ANNOVAR package (Version:2013-05-20) to annotate the SNPs.

Whole-genome resequencing (WGS) can detect a large number of SNPs information through sequence alignment. Correlations between the SNPs and traits were tested using mixed linear models in GEMMA software (13, 14). The statistical analysis model used in this study was*y* = *Xβ*+*Zγ*+*Wδ*+*e*, where y is the phenotypic trait, *Xβ* is the matrix of the fixed effects, which include gender, the first three principal components, variety, and field. *Zγ* is a matrix of random effects, γ represents random effects ($Var\left(\gamma \right)={\sigma_{g}}^{2}K$, *K* is the kinship matrix). *Wδ* is a matrix of random effects, and e is the random error with the distribution e~N (0, *Iσe*^2^), among them I is the identity matrix, σ*e*^2^ is the unknown residual. Population genetic structure and sex were used as fixed effects, and individual kinship was used as a random effect to correct for the influence of population structure and individual kinship. The significance threshold for GWAS was defined using the Bonferroni correction method. The total type I error rate was controlled at 5%, and the significance threshold of the genome was 0.05/Nsnp, where Nsnp is the number of SNPs remaining after quality control (15).

### SNP site screening principle and probe design

To meet the requirements of chip development, the position information of the target site and the 200bp sequence upstream and downstream of the site were compared with the reference genome Oar_v4.0 (GCF_000298735.2) using blastn (parameter: value 1e^−5^) (16). The accuracy of the site location information was checked by selecting the comparison result with the highest score according to the score value of the comparison. After the accuracy of the target site position information was verified, the probes were designed using Geno Baits Probe Designer software, with probe length set at 110 nt and GC content >30%. Every SNP site covered by at least three liquid-phase capture probes. Probes selected with GC content between 30% and 80%; Calculate the number of homology regions, and then pick the probes with homology regions < 10; The selected region should not contain probes in SSR or N regions; Filter out the final probe sequence to confirm the coverage of the site (probe design parameters: -len 110–110-gc 30–80-hom 5–d 2 -size 120–dis 20). Biotin-labeled polynucleotide probes were synthesized using *in situ* chip synthesis technology. Subsequently, these probes hybridize with target genomic regions to form double strands. Streptavidin-coated magnetic beads then adsorb biotinylated molecules. Following elution, amplification, and sequencing, SNP genotypes are identified.

### Performance evaluation of the 1K liquid chip

Four hundred and three samples were used for DNA library construction and hybridization capture sequencing to test the 1K liquid chip. Raw data obtained by sequencing were filtered using fastp (version 0.20.0, parameter: -n 10 -q 20 -u 40) to remove the adapter sequence and paired reads containing N content more than 10% and low-mass (Q ≤ 20) bases that exceed 40%. The BWA software (MEM comparison method) (v0.7.11) (10) was used to compare with the reference genome sequence (Oar_v4.0, GCF_000298735.2). The BAM files were sorted using Samtools software (v1.18) (11) and then removed duplicate sequences using GATK software (v4.1.4.1) (17).

The quality of the targeted sequencing data was assessed using Picard's CollectHsMetrics (18) was used to calculate metrics including Fold-80 penalty and the fraction of target bases achieving at least 10 × coverage.

GCTA (v1.92.2) was used to conduct principal component analysis (PCA) of the SNPs in all samples (19). Haploview was used to calculate the LD size (r2) between two pairs of markers and to plot the change with distance (20). NJ tree was constructed using MEGA-X software (model: p-distance; bootstrap: 1,000 times) (21). Population structure analysis was performed using Admixture (v1.3) (22), assuming that the number of groups (K value) of the sample was 1–15, and then clustered according to the Cross-Validation Error (CV error); the smallest K value corresponded to the optimal number of groups. TASSEL was used to calculate common GWAS models (GLM, GLM(Q), MLM(K), and MLM(QK) models) (23). The population structure matrix corresponding to the optimal K value from Admixture was used as the Q matrix of the corresponding model, and the inter-sample affinity matrix calculated using gcta software was used as the K matrix of the corresponding model. The *p*-value obtained by the calculation was -log10 and displayed in the Manhattan and Q-Q plots.

## Results

### Design of the 1K liquid chip

**Figure 1:**
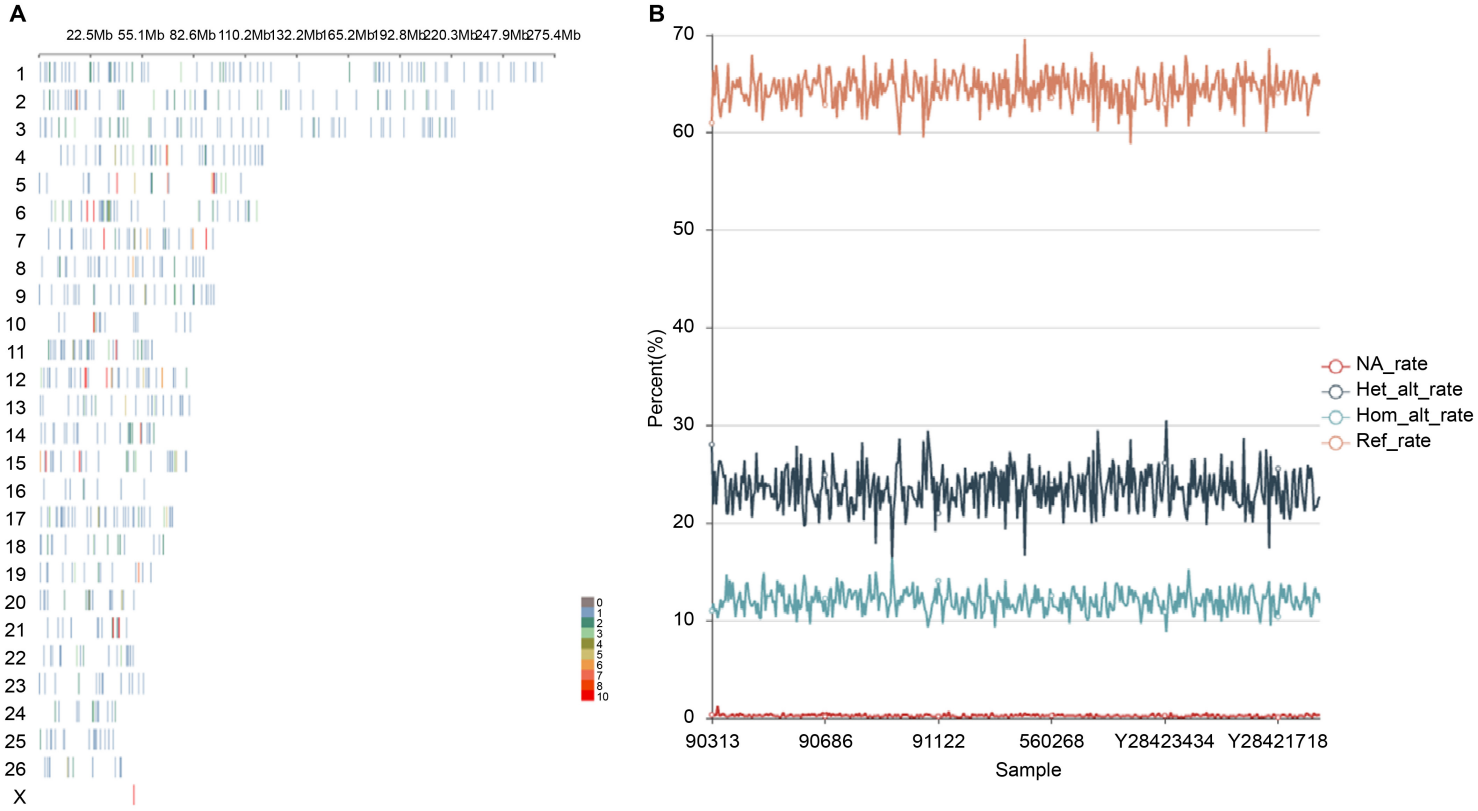
Sites of 1K liquid chip. **(A)** Location map of chromosome distribution. **(B)** Sample statistic. NA_rate ≤ 5% is the sample detection miss rate meeting the requirements; the lower the NA_rate, the fewer the number of undetected sites in the sample.

A total of 988 functional markers related to wool traits, 672 functional markers related to weight traits, 301 integrated functional markers related to important economic traits of fine wool sheep reported in the literature and OMIA (Online Mendelian Inheritance in Animals), and 1,961 functional labels were used in this study. After site checking, conversion of reference genome and data cleaning (format adjustment, removal of duplicate sites, removal of sites located on chromosome 27), 1,875 sites remained. For 620 samples as research group, A total of 722 sites with minor allele frequency (MAF) < 0.05, missing rate >20%, and non-binary sites with heterozygosity ratio >80% were removed. Finally, 1,012 SNPs were retained after probe design and screening, and the probe design coverage was 63.36%. The 1,012 SNPs distribution plots of the probe design are shown (site distribution parameter w 500000-n 1) (Figure 1A). After the synthesis of the functional marker probe was completed, six out of 409 samples were randomly selected for testing, and the final sample site capture efficiency was between 99.07% and 99.58%. Probe synthesis to identify functional markers has been successful, and the site capture efficiency can be used for large-population genotyping.

### Genotyping performance of the 1K liquid chip

All 403 samples were sequenced using the 1K liquid chip yielded satisfactory results. After filtering, a total of 573,482,034 clean reads were obtained from 578,704,792 raw reads. A total of 86,805,718,800 bp of raw bases was obtained, and 82,796,415,340 bp of clean bases were obtained after filtration, with an effective rate of 91.58–96.74%, Q20 of 97.05–98.64%, and Q30 of 90.1–95.37%. Homogeneity and on-target rate jointly determine the efficiency of targeted sequencing. The uniformity of coverage and capture efficiency were quantified, with the Fold-80 penalty at 2.3 and the fraction of targets covered≥10 × at 99.24%. A total of 505,741,155 alignments were matched to the reference sequence across all clean reads, with a maximum sample alignment rate of 90.33% and a minimum rate of 86.85%. There were 25 samples with missing sites >5, accounting for 6% of the total sample size. The deletion rate at this site was between 0.08% and 1.27%. The number of heterozygous sites ranged from 189 to 359, and the heterozygosity rate ranged from 16.07% to 30.53%. Furthermore, the number of homozygous sites was 104–193, whereas the heterozygous rate ranged from 8.84% to 16.41%. The number of functional sites consistent with the reference sequence in the sample ranged from 692 to 816, and the ratio of the number of functional sites consistent with the reference sequence to the total number of non-missing sites ranged from 58.84 to 69.62 (Figure 1B).

### Application of the 1K liquid chip

#### Marking quality control

Rare alleles, high deletion rates, and high heterozygosity alleles can cause abnormalities in population analysis and GWAS; therefore, this study filtered the marker sites in the original data and removed sites with minor allele frequencies (MAF) of < 0.05, sites with deletion rates >20%, sites with heterozygous ratios >80%, and non-binary allele sites. Finally, 1012 SNPs were labeled and filtered, and 722 SNPs were retained for subsequent analysis.

#### Principal component analysis and phylogenetic tree construction

**Figure 2:**
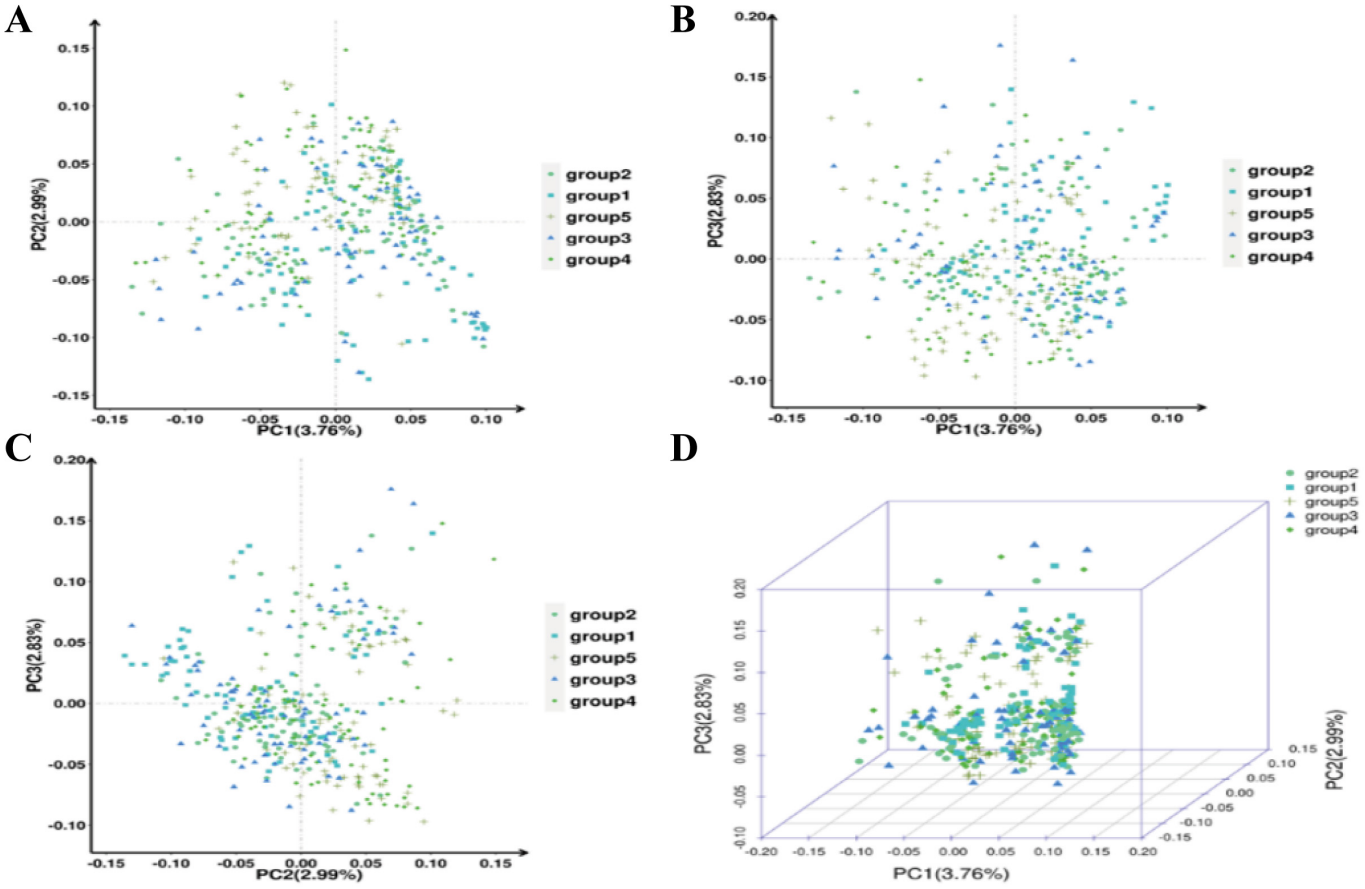
**(A–D)** PCA sample clustering diagram.

**Figure 3:**
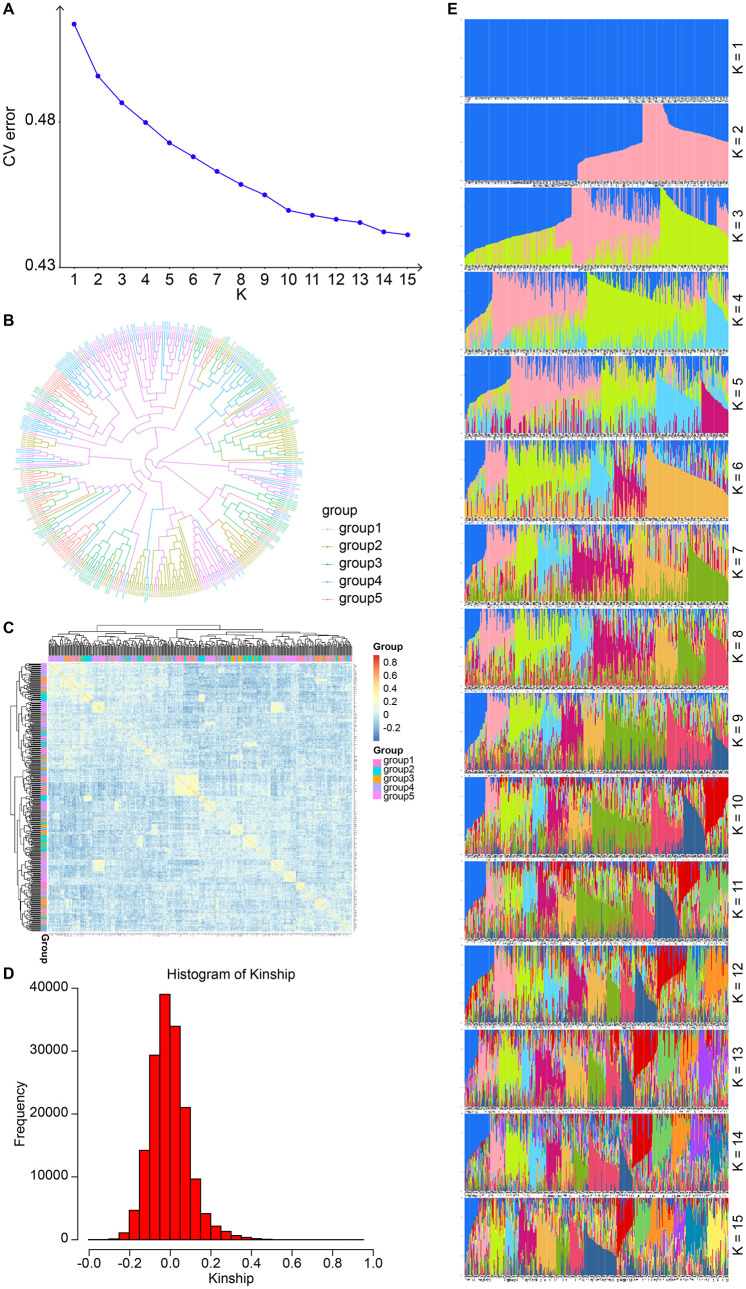
Population structure analysis. **(A)** CV error. **(B)** Phylogenetic tree. **(C)** Kinship heat map. **(D)** Frequency distribution of kinship relationships. **(E)** Histogram of the genetic composition of samples with different numbers of subgroups.

Based on the filtered 722 SNP markers, this project conducted principal component analysis (PCA) to obtain the variance interpretation rate of each principal component (PC) and the score matrix of the sample in each PC. Key information extracted from the SNP information was divided into PC1, PC2, PC3, etc., according to the effects from large to small, and the scatter plot of the first three PCs pairwise is shown in Figure 2. The variance interpretation rates for PC1, PC2, and PC3 were 3.76%, 2.99%, and 2.83%, respectively. This study fully utilized the 1K liquid chip sequencing data from 403 AMS to construct a phylogenetic tree (Figures 3A, B) and population structure (Figures 3C–E), indicating relatively close genetic distances.

#### Genome-wide association analysis

**Figure 4:**
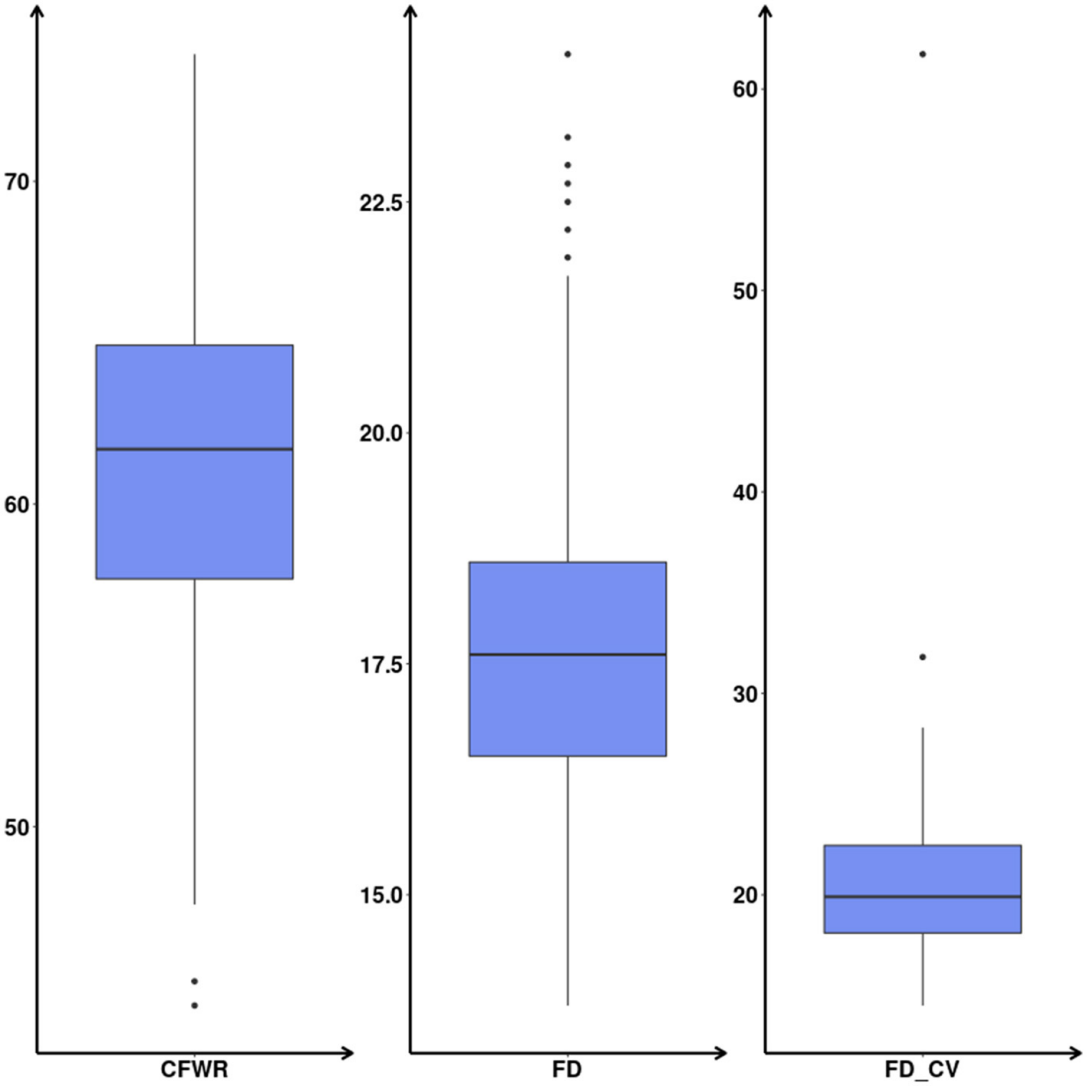
Boxplot of phenotypic values.

Before conducting GWAS, the phenotypic data were tested for extreme values, and the 3δ rule was used to eliminate phenotypic extremes, i.e., extremes outside the range of plus or minus three standard deviations of the mean value were eliminated. After calculating the phenotypic data used in this analysis according to the above rule, no abnormal samples were found for each trait. The descriptive statistics of the phenotypic values are shown in Table 1 and Figure 4.

**Table 1:** Descriptive statistics of phenotypic values.

**Trait**	***N***	**Mean**	**SD**	**Median**	**Trimmed**	**Mad**	**Min**	**Max**	**Range**	**Skew**	**Kurtosis**	**SE**
CFWR	403	61.07464	5.22439	61.7	61.27127	5.33736	44.46	73.94	29.48	−0.3738	−0.34349	0.26025
FD	403	17.69801	1.65717	17.6	17.61084	1.63086	13.8	24.1	10.3	0.57052	0.66058	0.08255
FD_CV	403	20.43382	3.59559	19.9	20.22817	3.11346	14.5	61.73	47.23	3.92892	41.42379	0.17911

**Figure 5:**
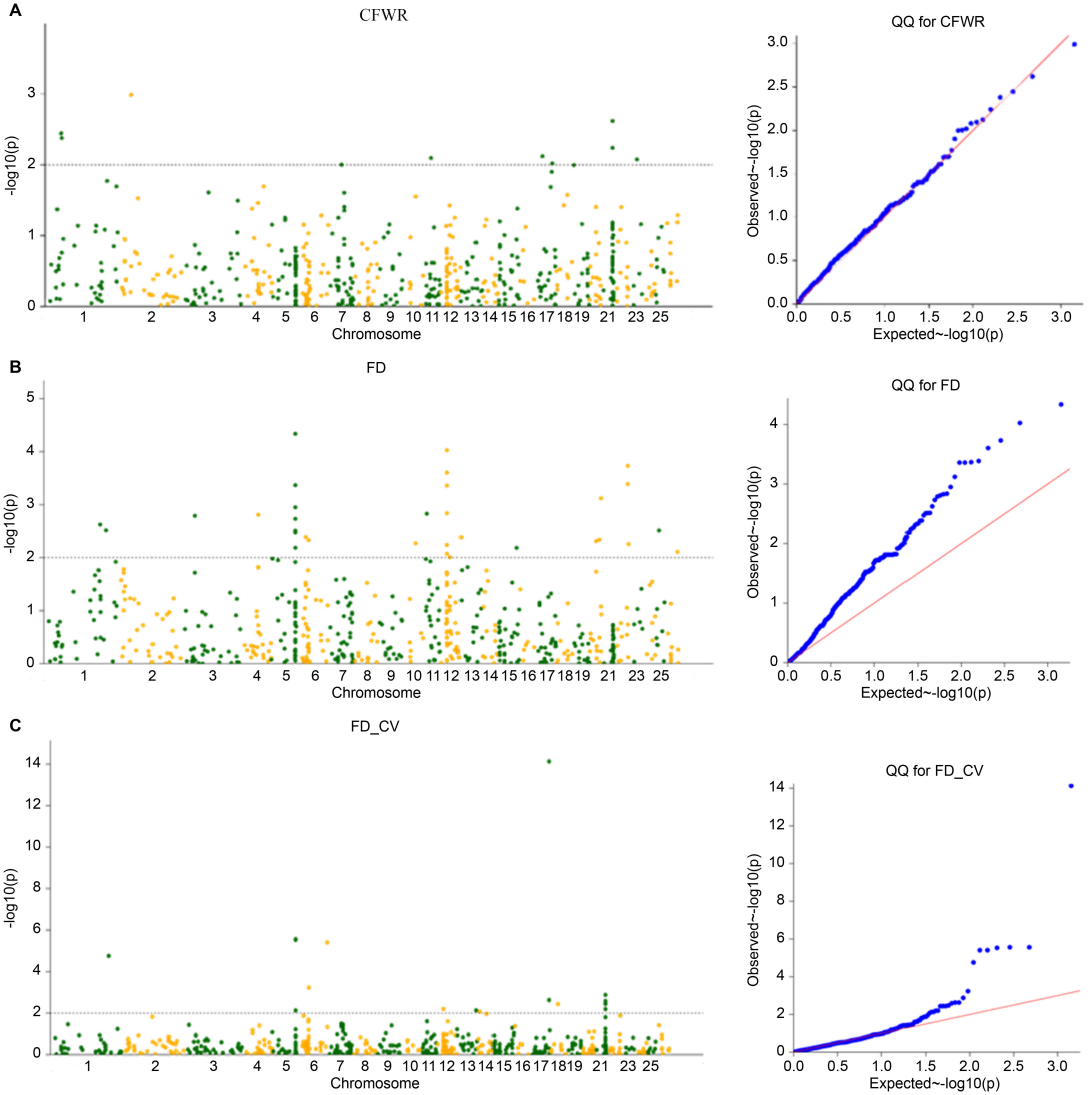
**(A–C)** GLM model Manhattan and QQ charts.

**Figure 6:**
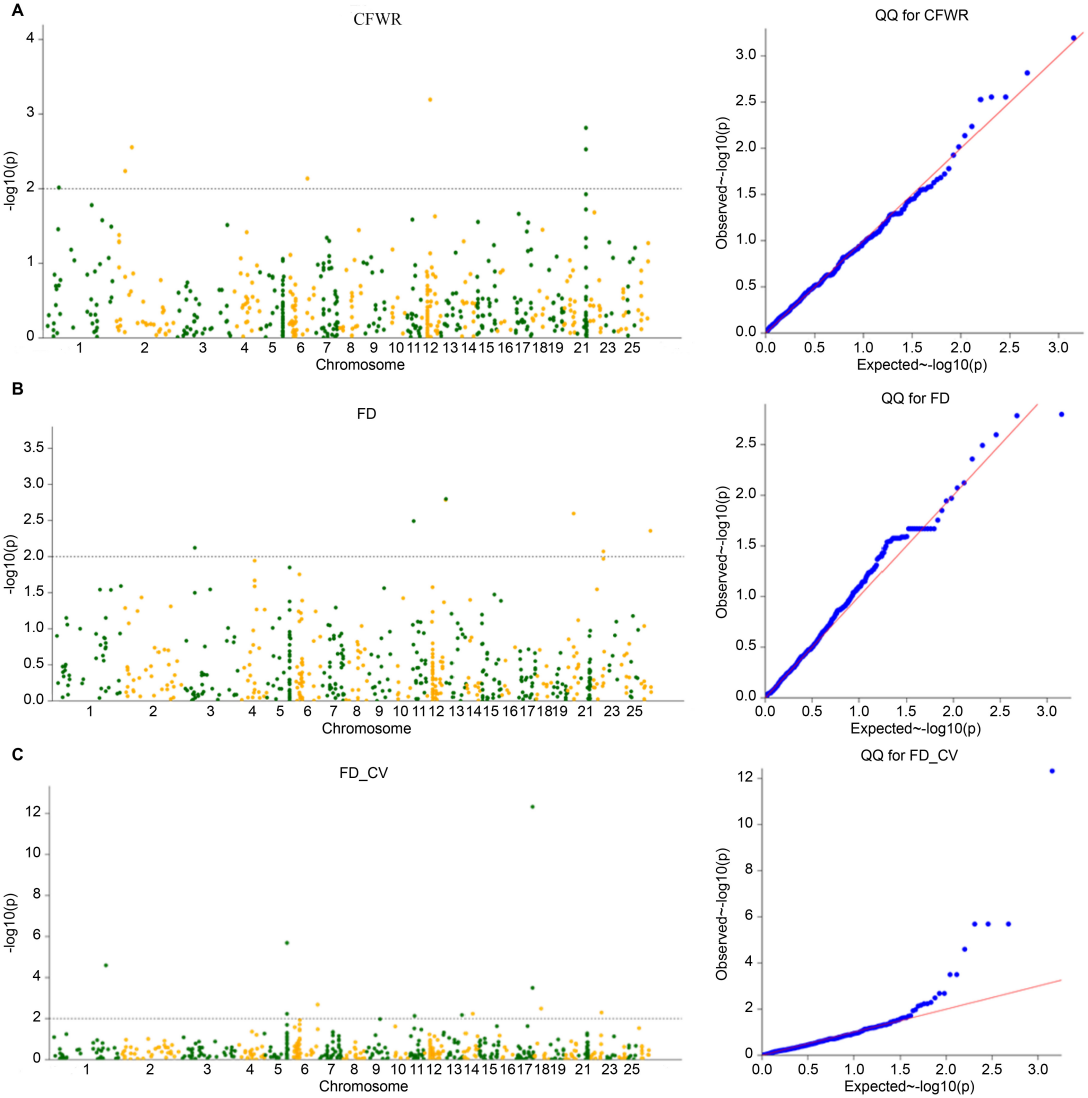
**(A–C)** GLM (Q) model Manhattan and QQ charts.

**Figure 7:**
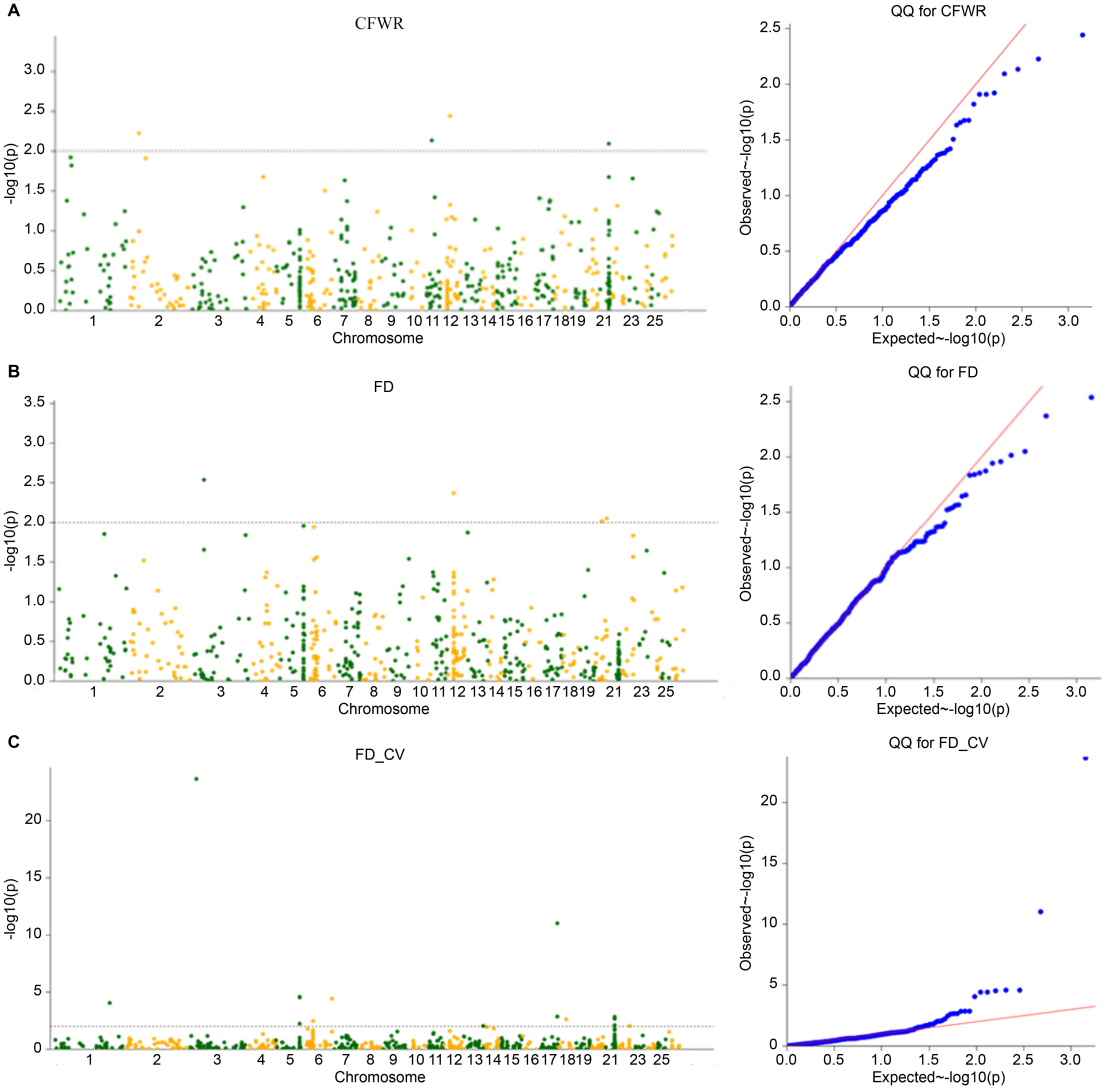
**(A–C)** MLM (K) model Manhattan and QQ charts.

**Figure 8:**
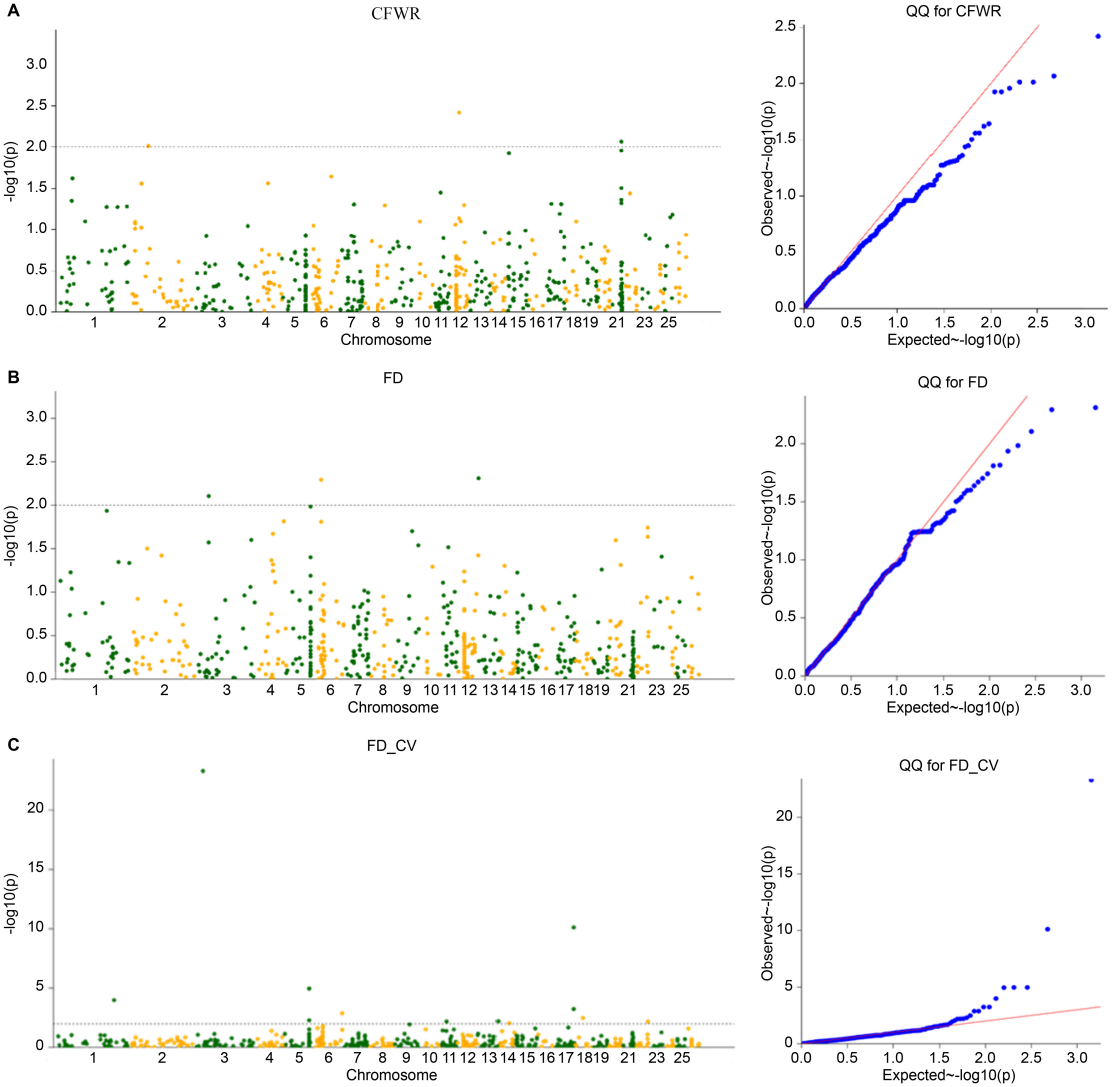
**(A–C)** MLM (QK) model Manhattan and QQ plots.

The four models commonly used in GWAS models (GLM, GLM(Q), MLM(K), and MLM(Q) models) were calculated using TASSEL software. The population structure matrix corresponding to the optimal K value of Admixture was used as the Q matrix of the corresponding model, and the inter-sample affinity matrix calculated by the gcta software was used as the K matrix of the corresponding model. The *p*-value obtained by the calculation was -log10 and Manhattan and Q-Q plots are plotted (Figures 5A–7C). The four models obtained significant SNP numbers related to cleaning rate, wool fiber diameter, and wool fiber diameter coefficient of variation of 27, 47, and 71, respectively, and the number of candidate genes was 30, 49, and 60, respectively, after annotation (Table 2, Figures 5–8).

**Table 2:** Significant SNPs and genes in different models.

**Model**	**CFWR**		**FD**		**FD-CV**	
	**SNPs**	**Genes**	**SNPs**	**Genes**	**SNPs**	**Genes**
GLM	11	14	33	30	21	15
GLM (Q)	8	7	7	10	15	13
MLM (K)	4	6	4	5	19	17
MLM (QK)	4	3	3	4	16	15

A total of 67 candidate genes were annotated based on the 100 kb region upstream and downstream of the significantly correlated SNP site, including 12 candidate genes related to fiber diameter, namely *BICC1, CAPN2, ELOVL2, ELOVL*5, *ERAP1, ERAP2, JAZF1, MFSD1, PLCB1, RNF144B, RNF43*, and *UBE2E1*. There were 13 candidate genes associated with the coefficient of variation of fiber diameter, which included *CDH1, CAPN2, CDH1, CRABP1, CRYBA4, DDX27, FADS2, IREB2, LCORL, LDB2, METTL16, SDR42E1*, and *WDR49*. Twelve candidate genes were associated with net gross rate, which included *LZTS1, MYO1E, RABEP1, MGST2, SRRM4*, S*UDS3, SLC1A1, EGFR, FADS2, RBBP8, PRRX1*, and *GRSF1*.

**Figure 9:**
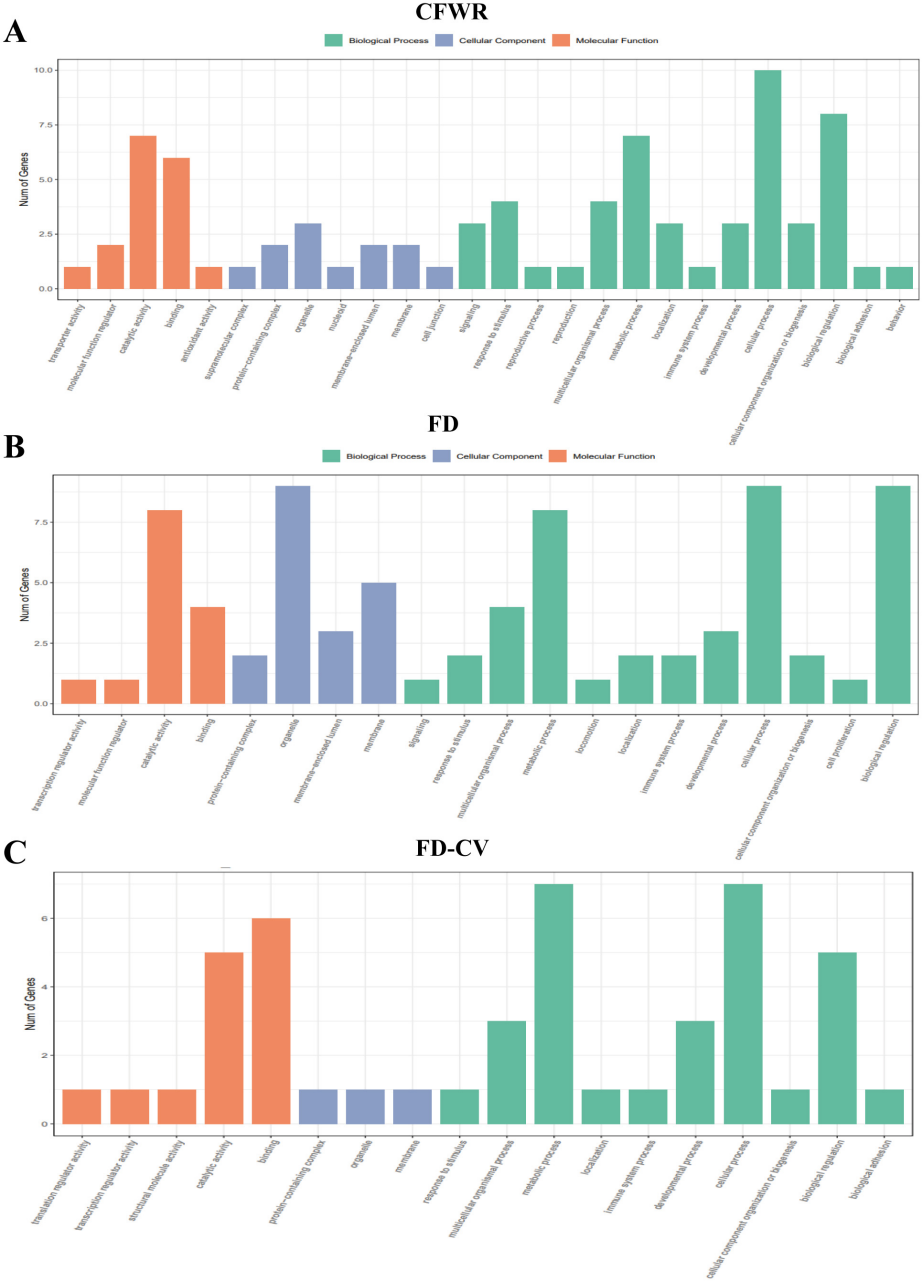
**(A–C)** GO analysis of candidate genes.

The results of GO enrichment analysis on these candidate genes showed that the main enriched biological processes included carboxylic acid metabolism, cell response to nitrogen compounds, cell response to organic nitrogen compounds, cell response to oxygen-containing compounds, embryo development, lipid catabolic process, organic acid metabolism process, oxygen acid metabolism process, response to nitrogen compounds, response to organic nitrogen compounds, and oxygen acid metabolism process. The cellular components have membrane microdomains and perinuclear regions of the cytoplasm, and the molecular function is lipid binding (Figure 9).

The results of KEGG pathway enrichment showed that they were mainly enriched in biosynthesis of unsaturated fatty acids, α-linolenic acid metabolism, cell adhesion molecules, Rap1 signaling pathway, PPAR signaling pathway, HIPPO signaling pathway, Apelin signaling pathway, apoptosis, cellular senescence, GnRH signaling pathway, and gap junction. The pathways enriched in all three traits were biosynthesis of unsaturated fatty acids, adhesion, and the Rap1 signaling pathway.

## Discussion

In recent years, molecular marker technology has undergone a series of revolutionary changes in terms of the quantity, variety, throughput, and cost of analysis (24). International multinational breeding companies have long regarded molecular breeding and its close integration with conventional breeding techniques as important modern breeding methods. Large breeding companies have a complete industrial chain and market share that can support high R&D investment and establish platforms, technologies, and methods that efficiently use molecular marker detection systems to support their breeding processes. However, small- and medium-sized breeding companies cannot establish efficient and low-cost molecular marker-assisted breeding systems, which has become an important bottleneck restricting molecular breeding (25).

One of the difficulties with genomic selection is the cost of genotyping, although with the development of microarray technology and high-throughput sequencing, the cost has been greatly reduced. However, in dairy cows, because of their high individual value and long era interval, the cost of typing is acceptable relative to the individual value of dairy cows; however, for species with small individual value and short era interval, it still means high cost and lacks sufficient economic benefits. The use of medium- to high-density genotyping chips in sheep has enabled genomic selection in sheep breeding since 2009, but the cost of genotyping remains high for many routine uses. Therefore, it is necessary to develop low-density genotyping chips to reduce costs. One way to solve this problem is to perform genotyping using low-density chips and then infer and “fill” in the missing SNPs from high-density chips or resequencing data (26). Filling can only produce effective results if it is based on a low recombination rate within one generation and repeated every three to four generations. A better filling effect can only be achieved when the number of SNPs is increased. However, when a higher number of SNPs on a low-density SNP chip leads to an increase in the number of genotypes used to identify the corresponding reference haplotype, the probability of randomly identifying haplotypes common to the reference and candidate populations decreases (27, 28). The 1K Liquid Chip of fine wool sheep developed in this study can provide a tool and technical instruments for low-cost, large-scale implementation of fine wool sheep genome selection.

GWAS technology has identified multiple genetic variants associated with weight, disease resistance, meat quality, and hairy traits in sheep, but only a few SNP loci sets significantly related to important economic traits of fine wool sheep have been reported. The SNP locus set significantly related to the important economic traits of fine wool sheep obtained in this study was screened from resequencing data by GWAS, and then collected from other important functional sites reported in the literature to form a 1K liquid chip related to important economic traits of fine wool sheep. To more accurately determine the SNPs related to fiber diameter, fiber diameter coefficient of variation, and cleaning rate of fine wool sheep, this study also used the 1K liquid chip of fine wool sheep to perform GWAS on AMS, and some clearer and more accurate important SNPs and functional genes were mined, indicating that the collection can be used for genome association analysis and candidate functional gene mining.

GWAS accuracy depends on the phenotypic accuracy employed in the analysis and the degree of computational model fit. In this study, two models, GLM and MLM, the common models of GWAS, were used for calculation, the population structure matrix corresponding to the optimal K value of Admixture was used as the Q matrix of the corresponding model, and the inter-sample affinity matrix calculated by gcta software was used as the K matrix of the corresponding model. GLM is a fixed-effect model, and MLM adds random effects, that is kinship matrices, in addition to fixed effects. The focus of GWAS is to mine significant loci; while the focus is on fixed effects, random effects are added to control false positives at sites that are significantly correlated with phenotype. In order to increase the accuracy and credibility of candidate gene mining, two models were used and different effects were added to perform four calculations. Finally, the results of the four models were intersected to obtain candidate genes related to the fiber diameter, fiber diameter coefficient of variation, and cleaning rate of fine wool sheep.

In this study, candidate genes *FADS2, FADS3, TDRD15, CDH1, CRABP1, CRABP4*, and *DDX27* were annotated in wool fiber diameter and cleaning rate, and it was found that these candidate genes were closely related to biological mechanisms such as skin development, defense, regeneration, and proliferation. For example, *FADS2* is very important for human skin, and dysfunction of sebaceous FADS2 activity may play a role in skin abnormalities associated with skin lipids (29). Studies have shown that branched-chain fatty acids (BCFAs) and straight/normal odd-chain fatty acids (n-OCFAs) are the main fatty acids in human skin lipids, especially in sebaceous gland (SG) wax esters. Skin lipids contain varying amounts of monounsaturated BCFAs and n-OCFAs, accounting for >20% of the total fatty acids in some reports. Fatty acid desaturase 2 (FADS2) encodes a versatile enzyme that catalyzes the desaturation of Δ4-, Δ6-, and Δ8-to 10 polyunsaturated fatty acids, but only one saturated fatty acid palmitate, converting it to 16:1n-10; FADS2 is inactive near 14:0 or 18:0 (30). TDRD15 was found to be significantly upregulated in the skin of patients with atopic dermatitis (AD) in comparison with the proteomic outcomes of normal hair follicles in patients with atopic dermatitis (31). AD, a common inflammatory skin disease characterized by skin and systemic inflammation as well as barrier dysfunction, suggests that the upregulation of the conserved tudor domain protein 15 (TDRD15) involves complex biological processes such as skin hydration processes, protease-proteasome complex interactions, lipid metabolism, antioxidants, and imbalances in inflammatory pathways. E-cadherin (CDH1) is a well-known cell-to-cell adhesion molecule that mechanically stretches to promote skin cell regeneration and proliferation. Mechanical stress is transmitted to the epidermis and induces downstream signaling pathways to induce epidermal cell differentiation, and CDH1-induced keratinocytes dedifferentiation is a key event in mechanical stretching-mediated skin regeneration. *CDH1* can be used as a potential therapeutic target to promote skin regeneration (32). CRABP1 is dynamically expressed as a cell-binding protein for retinoic acid (RA) signaling during skin development and in adult tissues (33). Although RA signaling is essential for epidermal differentiation, the mechanism of action is unknown and the distribution of RA between different nuclear receptors is regulated by RA-binding proteins. *CRABP1* is expressed in the stroma of embryonic dermis and skin tumors but limited to the follicular dermal papilla of normal postnatal skin, which is upregulated in response to RA treatment or Notch activation and is negatively regulated by Wnt/β-catenin signaling. In this study, candidate genes *CRABP1* and *CRABP4* were also identified, indicating that they are involved in the biological function of fine wool sheep skin tissue development and the key pathway of wool growth. Other skin-related candidate genes, such as *DDX27*, were also found in this study, and it was reported that *DDX27* is related to pathways associated with the hyperpigmentation of chicken skin through genetic annotation using selective signal analysis (34). *ELOVL5* is prominently expressed in the basal layer of sebaceous gland cells in the head skin of male goats, and the candidate genes for pheromone synthesis are mainly expressed in the sebaceous glands of the pheromone-producing skin region. The “male effect” is a well-known phenomenon in female sheep and goats that occurs due to pheromone-induced activation of reproductive function, and studies have found significant increases in gene expression in the extended long-chain fatty acid family member 5 (*ELOVL5*) while inducing pheromone synthesis; therefore, *ELOVL5* is considered to be the main candidate gene for pheromone synthesis (35).

## Conclusions

In this study, 1,012 SNPs associated with economically important traits in fine wool sheep were screened from resequencing data using GWAS and pooled into a 1K liquid chip for genome-wide association analysis. Four models yielded 145 significant SNPs, which were annotated to 139 candidate genes associated with the washing ratio, wool fiber diameter, and coefficient of variation of wool fiber diameter. GO and KEGG enrichment analyses showed that the biosynthesis of unsaturated fatty acids, adhesion, and the Rap1 signaling pathway were enriched in all three traits.

## Data Availability

The original contributions presented in the study are publicly available. This data can be found here: BioProject: PRJNA1162637, https://www.ncbi.nlm.nih.gov/bioproject/?term=PRJNA1162637.
