# Supplementary material for: Design, validation, and application of a 1K liquid chip for genome-wide association analysis in Alpine Merino sheep
Source: Front Vet Sci. 2025 Dec 18;12:1690580. doi: 10.3389/fvets.2025.1690580 (PMC12756972; doi:10.3389/fvets.2025.1690580)
Supplement: Supplementary file 1 [file Table_1.docx]

***Supplementary Material***

1. **Supplementary Tables**

# **Supplement Table 1 1012 SNPs position information**

| **No.** | **Chromosome** | **Location** | **Genotype** | **No.** | **Chromosome** | **Location** | **Genotype** |
| --- | --- | --- | --- | --- | --- | --- | --- |
| 1 | 1 | 3451931 | A/- | 21 | 1 | 49921099 | C/G |
| 2 | 1 | 5861251 | G/A | 22 | 1 | 54840055 | G/T |
| 3 | 1 | 5862100 | T/G | 23 | 1 | 56184572 | G/T |
| 4 | 1 | 8622748 | C/G | 24 | 1 | 58096115 | A/G |
| 5 | 1 | 14430847 | G/C | 25 | 1 | 75928644 | A/G |
| 6 | 1 | 16077946 | G/A | 26 | 1 | 75928652 | A/G |
| 7 | 1 | 27037196 | G/T | 27 | 1 | 75929075 | C/T |
| 8 | 1 | 27037212 | A/C | 28 | 1 | 84199588 | T/C |
| 9 | 1 | 27726221 | T/C | 29 | 1 | 96944832 | G/C |
| 10 | 1 | 27807636 | T/G | 30 | 1 | 98462279 | C/A |
| 11 | 1 | 29018708 | G/A | 31 | 1 | 98462319 | C/T |
| 12 | 1 | 31970394 | T/G | 32 | 1 | 99739982 | G/A |
| 13 | 1 | 37405688 | C/T | 33 | 1 | 101961540 | A/T |
| 14 | 1 | 37524923 | A/G | 34 | 1 | 107253851 | T/G |
| 15 | 1 | 38279842 | C/T | 35 | 1 | 107960988 | G/A |
| 16 | 1 | 40229782 | T/A | 36 | 1 | 112836847 | T/G |
| 17 | 1 | 40694525 | C/T | 37 | 1 | 123500184 | C/G |
| 18 | 1 | 42790552 | T/C | 38 | 1 | 165696300 | T/C |
| 19 | 1 | 46959081 | T/C | 39 | 1 | 165698116 | G/T |
| 20 | 1 | 49920695 | G/A | 40 | 1 | 178644107 | C/T |
| 41 | 1 | 179554675 | G/A | 83 | 2 | 25369713 | A/G |
| 42 | 1 | 179765629 | G/C | 84 | 2 | 38882962 | T/A |
| 43 | 1 | 181701747 | A/G | 85 | 2 | 42422497 | A/G |
| 44 | 1 | 183906382 | A/G | 86 | 2 | 43428586 | G/T |
| 45 | 1 | 193784349 | T/G | 87 | 2 | 45125479 | G/A |
| 46 | 1 | 198584375 | A/G | 88 | 2 | 49481881 | A/G |
| 47 | 1 | 201761544 | C/T | 89 | 2 | 61249079 | C/G |
| 48 | 1 | 203825947 | T/A | 90 | 2 | 61291607 | A/G |
| 49 | 1 | 205430439 | T/G | 91 | 2 | 61291610 | T/A |
| 50 | 1 | 205462185 | T/C | 92 | 2 | 68604207 | T/G |
| 51 | 1 | 205478374 | C/T | 93 | 2 | 72373820 | C/T |
| 52 | 1 | 205500519 | C/A | 94 | 2 | 72377076 | A/G |
| 53 | 1 | 211543576 | T/A | 95 | 2 | 77090829 | T/C |
| 54 | 1 | 216530176 | A/G | 96 | 2 | 77662645 | A/G |
| 55 | 1 | 217783263 | C/G | 97 | 2 | 80650248 | G/T |
| 56 | 1 | 226733906 | C/T | 98 | 2 | 88988119 | G/A |
| 57 | 1 | 236304249 | A/T | 99 | 2 | 89058360 | A/G |
| 58 | 1 | 239376005 | C/T | 100 | 2 | 95799483 | G/A |
| 59 | 1 | 253204967 | T/C | 101 | 2 | 95799486 | C/T |
| 60 | 1 | 263544281 | T/C | 102 | 2 | 101770384 | T/C |
| 61 | 1 | 264549552 | C/G | 103 | 2 | 106614193 | G/A |
| 62 | 1 | 268085615 | C/G | 104 | 2 | 116558556 | G/T |
| 63 | 2 | 5882448 | C/A | 105 | 2 | 116670016 | A/G |
| 64 | 2 | 5882774 | T/C | 106 | 2 | 118577752 | T/C |
| 65 | 2 | 10198248 | C/T | 107 | 2 | 128841355 | G/A |
| 66 | 2 | 15695196 | A/G | 108 | 2 | 129025721 | C/T |
| 67 | 2 | 17226713 | A/G | 109 | 2 | 129025780 | G/A |
| 68 | 2 | 19053863 | T/A | 110 | 2 | 131586072 | G/T |
| 69 | 2 | 19990277 | C/T | 111 | 2 | 146293547 | C/T |
| 70 | 2 | 20056143 | G/A | 112 | 2 | 158726692 | A/G |
| 71 | 2 | 20098908 | A/G | 113 | 2 | 169145867 | G/T |
| 72 | 2 | 20098965 | A/G | 114 | 2 | 180632560 | C/T |
| 73 | 2 | 20100378 | T/C | 115 | 2 | 180657487 | T/C |
| 74 | 2 | 20100605 | T/C | 116 | 2 | 185219642 | C/G |
| 75 | 2 | 20103048 | G/A | 117 | 2 | 187118248 | C/A |
| 76 | 2 | 20106012 | C/G | 118 | 2 | 195082287 | T/C |
| 77 | 2 | 20142011 | T/C | 119 | 2 | 195124218 | C/A |
| 78 | 2 | 20245890 | A/C | 120 | 2 | 199978178 | A/G |
| 79 | 2 | 20249409 | C/T | 121 | 2 | 206527137 | T/C |
| 80 | 2 | 21539492 | C/T | 122 | 2 | 206565107 | C/A |
| 81 | 2 | 24981240 | G/A | 123 | 2 | 207622986 | T/C |
| 82 | 2 | 25347207 | T/G | 124 | 2 | 211547108 | G/C |
| 125 | 2 | 221384653 | T/C | 167 | 3 | 187402673 | T/C |
| 126 | 2 | 238608039 | T/C | 168 | 3 | 194880533 | A/G |
| 127 | 2 | 241834434 | A/T | 169 | 3 | 204589212 | T/C |
| 128 | 3 | 3556604 | A/G | 170 | 3 | 205721451 | A/G |
| 129 | 3 | 6714946 | A/G | 171 | 3 | 207766117 | G/A |
| 130 | 3 | 10578668 | C/A | 172 | 3 | 208938106 | T/C |
| 131 | 3 | 10608668 | C/A | 173 | 3 | 209796060 | C/G |
| 132 | 3 | 14108642 | T/C | 174 | 3 | 217112666 | G/A |
| 133 | 3 | 14108654 | T/C | 175 | 3 | 217456273 | C/T |
| 134 | 3 | 19115427 | G/A | 176 | 3 | 220453168 | G/T |
| 135 | 3 | 19115576 | T/G | 177 | 3 | 221933816 | G/A |
| 136 | 3 | 19115591 | A/G | 178 | 4 | 11592615 | G/A |
| 137 | 3 | 23503874 | A/G | 179 | 4 | 15445932 | A/T |
| 138 | 3 | 32157828 | G/A | 180 | 4 | 32255647 | C/T |
| 139 | 3 | 33473799 | G/A | 181 | 4 | 32622831 | T/C |
| 140 | 3 | 37784237 | C/T | 182 | 4 | 40519377 | T/G |
| 141 | 3 | 37788993 | G/A | 183 | 4 | 40519399 | T/C |
| 142 | 3 | 42170418 | C/T | 184 | 4 | 40519422 | A/G |
| 143 | 3 | 42193980 | T/C | 185 | 4 | 40875826 | C/A |
| 144 | 3 | 42768273 | C/T | 186 | 4 | 43606771 | G/A |
| 145 | 3 | 45341061 | G/T | 187 | 4 | 47273236 | C/T |
| 146 | 3 | 45470146 | A/C | 188 | 4 | 50168117 | C/T |
| 147 | 3 | 47028545 | T/C | 189 | 4 | 50182043 | G/A |
| 148 | 3 | 67494286 | T/C | 190 | 4 | 50301914 | A/G |
| 149 | 3 | 73816912 | G/A | 191 | 4 | 55894778 | C/T |
| 150 | 3 | 74005974 | G/A | 192 | 4 | 59570063 | C// |
| 151 | 3 | 75598278 | A/C | 193 | 4 | 60159529 | T/C |
| 152 | 3 | 75888377 | T/C | 194 | 4 | 62348329 | C/T |
| 153 | 3 | 81409321 | A/G | 195 | 4 | 62388564 | G/A |
| 154 | 3 | 83684556 | C/T | 196 | 4 | 68142771 | C/G |
| 155 | 3 | 95203288 | C/T | 197 | 4 | 68150060 | A/G |
| 156 | 3 | 102602387 | G/A | 198 | 4 | 68150136 | T/C |
| 157 | 3 | 108909221 | A/G | 199 | 4 | 68150568 | G/C |
| 158 | 3 | 117325341 | T/G | 200 | 4 | 68150631 | G/T |
| 159 | 3 | 122162033 | C/T | 201 | 4 | 68151156 | G/A |
| 160 | 3 | 146054056 | C/T | 202 | 4 | 68151188 | C/T |
| 161 | 3 | 146073597 | A/G | 203 | 4 | 68151519 | G/C |
| 162 | 3 | 146785479 | G/A | 204 | 4 | 68151699 | T/A |
| 163 | 3 | 146804020 | C/T | 205 | 4 | 68151727 | A/G |
| 164 | 3 | 156212588 | G/A | 206 | 4 | 68152565 | C/T |
| 165 | 3 | 163001279 | G/A | 207 | 4 | 68153060 | G/T |
| 166 | 3 | 177405156 | G/T | 208 | 4 | 68153189 | T/A |
| 209 | 4 | 68153218 | T/A | 251 | 5 | 41768607 | C/T |
| 210 | 4 | 68153287 | A/C | 252 | 5 | 41768682 | A/G |
| 211 | 4 | 68153294 | C/A | 253 | 5 | 41768701 | C/T |
| 212 | 4 | 68155041 | C/T | 254 | 5 | 41768845 | T/C |
| 213 | 4 | 68160427 | T/C | 255 | 5 | 41768883 | T/G |
| 214 | 4 | 68160841 | A/G | 256 | 5 | 41769002 | T/C |
| 215 | 4 | 68161213 | C/T | 257 | 5 | 41769076 | A/G |
| 216 | 4 | 68161248 | G/T | 258 | 5 | 41770341 | C/T |
| 217 | 4 | 68161562 | G/A | 259 | 5 | 41770576 | G/A |
| 218 | 4 | 68172566 | C/T | 260 | 5 | 51256416 | T/C |
| 219 | 4 | 68876516 | A/G | 261 | 5 | 51268810 | G/A |
| 220 | 4 | 68876654 | C/A | 262 | 5 | 51297757 | C/T |
| 221 | 4 | 68876686 | C/A | 263 | 5 | 51301134 | C/T |
| 222 | 4 | 68876697 | G/A | 264 | 5 | 51317143 | T/C |
| 223 | 4 | 68876717 | C/A | 265 | 5 | 59718004 | A/G |
| 224 | 4 | 76268884 | C/T | 266 | 5 | 60455332 | A/G |
| 225 | 4 | 85597532 | G/A | 267 | 5 | 68841519 | C/T |
| 226 | 4 | 87108792 | G/A | 268 | 5 | 68870654 | T/C |
| 227 | 4 | 88993708 | C/T | 269 | 5 | 68891936 | G/C |
| 228 | 4 | 88993776 | A/T | 270 | 5 | 68893691 | G/A |
| 229 | 4 | 89210903 | G/A | 271 | 5 | 68899917 | G/A |
| 230 | 4 | 92503453 | G/A | 272 | 5 | 68900527 | T/A |
| 231 | 4 | 98905740 | A/T | 273 | 5 | 68905390 | G/A |
| 232 | 4 | 106805132 | T/G | 274 | 5 | 69332305 | C/T |
| 233 | 4 | 109664158 | T/C | 275 | 5 | 72539245 | T/C |
| 234 | 4 | 112717393 | G/A | 276 | 5 | 77318265 | G/C |
| 235 | 4 | 117296554 | G/A | 277 | 5 | 92175736 | C/T |
| 236 | 4 | 118569623 | G/A | 278 | 5 | 92247625 | G// |
| 237 | 4 | 119215917 | C/T | 279 | 5 | 92256711 | T/C |
| 238 | 5 | 261484 | T/C | 280 | 5 | 92265355 | C/T |
| 239 | 5 | 4409988 | G/C | 281 | 5 | 92276610 | C/G |
| 240 | 5 | 23531093 | T/C | 282 | 5 | 92277630 | A/G |
| 241 | 5 | 26762581 | C/A | 283 | 5 | 93324254 | C/T |
| 242 | 5 | 41768121 | C/T | 284 | 5 | 93330606 | T/C |
| 243 | 5 | 41768179 | C/T | 285 | 5 | 93335425 | C/T |
| 244 | 5 | 41768260 | C/A | 286 | 5 | 93337944 | A/G |
| 245 | 5 | 41768355 | T/C | 287 | 5 | 93339683 | T/C |
| 246 | 5 | 41768437 | G/A | 288 | 5 | 93342367 | T/G |
| 247 | 5 | 41768473 | C/A | 289 | 5 | 93344478 | G/A |
| 248 | 5 | 41768505 | G/T | 290 | 5 | 93344882 | C/T |
| 249 | 5 | 41768511 | G/T | 291 | 5 | 93345980 | A/G |
| 250 | 5 | 41768541 | G/A | 292 | 5 | 93346515 | G/A |
| 293 | 5 | 93349994 | G/A | 335 | 6 | 25952803 | T/C |
| 294 | 5 | 93351608 | G/C | 336 | 6 | 25954256 | G/C |
| 295 | 5 | 93367693 | A/T | 337 | 6 | 25954677 | T/C |
| 296 | 5 | 93383288 | G/A | 338 | 6 | 25956512 | A/G |
| 297 | 5 | 93384120 | G/A | 339 | 6 | 25956668 | A/G |
| 298 | 5 | 93385396 | T/C | 340 | 6 | 25956686 | C/T |
| 299 | 5 | 93387255 | C/T | 341 | 6 | 25997419 | C/T |
| 300 | 5 | 93388756 | G/C | 342 | 6 | 29298893 | G/T |
| 301 | 5 | 93391985 | G/A | 343 | 6 | 29302663 | C/T |
| 302 | 5 | 93392877 | C/T | 344 | 6 | 29313254 | C/T |
| 303 | 5 | 93393426 | A/G | 345 | 6 | 29313279 | T/C |
| 304 | 5 | 93397780 | T/C | 346 | 6 | 29314362 | C/A |
| 305 | 5 | 93398765 | C/T | 347 | 6 | 29314392 | T/C |
| 306 | 5 | 93399780 | G/A | 348 | 6 | 29314656 | C/T |
| 307 | 5 | 93404769 | T/C | 349 | 6 | 29315643 | T/C |
| 308 | 5 | 93406719 | C/T | 350 | 6 | 29315891 | C/T |
| 309 | 5 | 93410192 | G/A | 351 | 6 | 29317926 | T/C |
| 310 | 5 | 93410812 | A/G | 352 | 6 | 29318943 | A/C |
| 311 | 5 | 93412906 | T/C | 353 | 6 | 29319153 | A/G |
| 312 | 5 | 93413296 | C/T | 354 | 6 | 29319460 | C/T |
| 313 | 5 | 93507537 | A/C | 355 | 6 | 29320736 | A/C |
| 314 | 5 | 97054949 | G/A | 356 | 6 | 29322402 | G/A |
| 315 | 5 | 97141788 | T/C | 357 | 6 | 29334836 | C/T |
| 316 | 5 | 97141851 | G/C | 358 | 6 | 29346692 | C/T |
| 317 | 5 | 99874827 | T/A | 359 | 6 | 32050951 | T/C |
| 318 | 5 | 99874842 | A/G | 360 | 6 | 36127367 | T/C |
| 319 | 5 | 99876420 | T/C | 361 | 6 | 37216183 | T/C |
| 320 | 5 | 107834012 | T/G | 362 | 6 | 37769711 | G/A |
| 321 | 6 | 6981182 | C/T | 363 | 6 | 40485673 | A/C |
| 322 | 6 | 8773737 | C/A | 364 | 6 | 41532048 | G/A |
| 323 | 6 | 8774526 | G/A | 365 | 6 | 49462644 | C/T |
| 324 | 6 | 8777453 | C/T | 366 | 6 | 66557778 | C/T |
| 325 | 6 | 12728357 | A/C | 367 | 6 | 85018210 | C/T |
| 326 | 6 | 12728434 | G/A | 368 | 6 | 85018830 | C/A |
| 327 | 6 | 16438006 | G/A | 369 | 6 | 85749376 | T/C |
| 328 | 6 | 16441499 | G/A | 370 | 6 | 92912924 | G/A |
| 329 | 6 | 16495709 | T/C | 371 | 6 | 92936288 | A/G |
| 330 | 6 | 17410955 | G/C | 372 | 6 | 106179116 | A/G |
| 331 | 6 | 22837362 | A/C | 373 | 6 | 110363000 | T/C |
| 332 | 6 | 25944136 | A/T | 374 | 6 | 111748267 | T/G |
| 333 | 6 | 25947038 | T/C | 375 | 6 | 111748269 | T/A |
| 334 | 6 | 25952072 | T/A | 376 | 6 | 112203175 | T/C |
| 377 | 6 | 116327927 | A/G | 419 | 7 | 82357379 | A/G |
| 378 | 6 | 116331212 | T/C | 420 | 7 | 82407479 | G/A |
| 379 | 6 | 116352111 | A/G | 421 | 7 | 82407496 | C/T |
| 380 | 7 | 5056143 | T/C | 422 | 7 | 82489749 | A/G |
| 381 | 7 | 6394501 | G/A | 423 | 7 | 82493823 | G/A |
| 382 | 7 | 17494202 | T/C | 424 | 7 | 89226421 | C/T |
| 383 | 7 | 17560390 | C/T | 425 | 7 | 89289232 | G/A |
| 384 | 7 | 23948568 | G/A | 426 | 7 | 89310845 | C/T |
| 385 | 7 | 25100215 | G/A | 427 | 7 | 89321251 | G/A |
| 386 | 7 | 27907651 | A/T | 428 | 7 | 89369706 | G/A |
| 387 | 7 | 34733888 | A/C | 429 | 7 | 89369939 | G/T |
| 388 | 7 | 34736705 | C/T | 430 | 7 | 89370119 | C/T |
| 389 | 7 | 34737813 | C/T | 431 | 7 | 89370278 | C/G |
| 390 | 7 | 34738236 | C/T | 432 | 7 | 89444586 | G/A |
| 391 | 7 | 34744386 | G/C | 433 | 7 | 92837555 | G/A |
| 392 | 7 | 34747476 | C/T | 434 | 8 | 1545545 | G/A |
| 393 | 7 | 34760953 | C/T | 435 | 8 | 11106076 | C/T |
| 394 | 7 | 34762250 | C/T | 436 | 8 | 11136076 | C/T |
| 395 | 7 | 34767069 | A/G | 437 | 8 | 18684032 | G/A |
| 396 | 7 | 34771149 | C/A | 438 | 8 | 19023022 | G/A |
| 397 | 7 | 40783477 | C/A | 439 | 8 | 21104197 | T/C |
| 398 | 7 | 40784018 | G/A | 440 | 8 | 26420904 | C/T |
| 399 | 7 | 48114170 | G/A | 441 | 8 | 27995767 | T/C |
| 400 | 7 | 50659683 | A/G | 442 | 8 | 36203431 | C/T |
| 401 | 7 | 51069476 | G/A | 443 | 8 | 40830278 | C/T |
| 402 | 7 | 51283269 | T/C | 444 | 8 | 47738959 | C/G |
| 403 | 7 | 51297390 | G/T | 445 | 8 | 50278661 | C/A |
| 404 | 7 | 51298844 | C/G | 446 | 8 | 50284096 | T/C |
| 405 | 7 | 51299903 | G/A | 447 | 8 | 50288215 | A/G |
| 406 | 7 | 54196920 | G/A | 448 | 8 | 50437219 | A/G |
| 407 | 7 | 57499456 | G/A | 449 | 8 | 50473103 | T/C |
| 408 | 7 | 57526727 | C/T | 450 | 8 | 50477424 | G/A |
| 409 | 7 | 57725891 | A/G | 451 | 8 | 51691445 | G/A |
| 410 | 7 | 57777457 | C/T | 452 | 8 | 52702998 | A/G |
| 411 | 7 | 57799864 | A/T | 453 | 8 | 61192575 | C/A |
| 412 | 7 | 57803771 | C/T | 454 | 8 | 65694498 | T/A |
| 413 | 7 | 57836206 | C/T | 455 | 8 | 72156970 | A/G |
| 414 | 7 | 59021362 | T/A | 456 | 8 | 72156992 | T/G |
| 415 | 7 | 67004071 | C/T | 457 | 8 | 78855672 | A/G |
| 416 | 7 | 67004083 | G/A | 458 | 8 | 82239902 | A/C |
| 417 | 7 | 69408170 | T/C | 459 | 8 | 85005324 | A/G |
| 418 | 7 | 82353169 | G/A | 460 | 8 | 85626160 | T/C |
| 461 | 8 | 87576336 | G/A | 503 | 10 | 29446075 | C/T |
| 462 | 9 | 4405 | C/T | 504 | 10 | 29446204 | C/T |
| 463 | 9 | 13510909 | C/T | 505 | 10 | 29456858 | G/A |
| 464 | 9 | 15051070 | T/G | 506 | 10 | 29456891 | G/A |
| 465 | 9 | 18949237 | G/A | 507 | 10 | 29473629 | G/A |
| 466 | 9 | 19840980 | C/T | 508 | 10 | 29473669 | C/A |
| 467 | 9 | 21203947 | T/C | 509 | 10 | 29473680 | C/G |
| 468 | 9 | 29377019 | A/G | 510 | 10 | 29509083 | G/A |
| 469 | 9 | 29377852 | C/T | 511 | 10 | 29509123 | C/T |
| 470 | 9 | 48756828 | G/A | 512 | 10 | 29509134 | G/C |
| 471 | 9 | 50219798 | A/G | 513 | 10 | 29542888 | T/C |
| 472 | 9 | 56560366 | G/A | 514 | 10 | 30994368 | T/C |
| 473 | 9 | 56582306 | A/C | 515 | 10 | 32485355 | A/T |
| 474 | 9 | 56585278 | C/T | 516 | 10 | 32753367 | G/T |
| 475 | 9 | 56605885 | C/T | 517 | 10 | 50582780 | G/C |
| 476 | 9 | 57396716 | T/C | 518 | 10 | 51739659 | G/A |
| 477 | 9 | 61532615 | A/G | 519 | 10 | 52551820 | G/A |
| 478 | 9 | 67836865 | G/A | 520 | 10 | 73248937 | T/C |
| 479 | 9 | 72302537 | A/C | 521 | 10 | 80901397 | A/G |
| 480 | 9 | 72302569 | T/C | 522 | 11 | 5243442 | A/T |
| 481 | 9 | 72761980 | C/A | 523 | 11 | 5479317 | A/T |
| 482 | 9 | 72806896 | C/T | 524 | 11 | 6088087 | T/C |
| 483 | 9 | 75509088 | C/G | 525 | 11 | 7888366 | G/A |
| 484 | 9 | 82575312 | G/A | 526 | 11 | 8917643 | C/A |
| 485 | 9 | 82689856 | G/A | 527 | 11 | 12049183 | C/T |
| 486 | 9 | 89321670 | G/A | 528 | 11 | 13743090 | C/G |
| 487 | 9 | 90322720 | G/T | 529 | 11 | 18292250 | A/G |
| 488 | 9 | 91746914 | T/G | 530 | 11 | 18302509 | C/T |
| 489 | 9 | 93419159 | C/T | 531 | 11 | 18401243 | A/G |
| 490 | 10 | 10874376 | T/C | 532 | 11 | 18408552 | C/T |
| 491 | 10 | 13697933 | A/T | 533 | 11 | 18916974 | C/T |
| 492 | 10 | 29434962 | T/C | 534 | 11 | 19249538 | A/G |
| 493 | 10 | 29435388 | A/G | 535 | 11 | 21895872 | C/T |
| 494 | 10 | 29435511 | T/A | 536 | 11 | 22968968 | C/T |
| 495 | 10 | 29439011 | C/T | 537 | 11 | 25845229 | C/T |
| 496 | 10 | 29439053 | C/T | 538 | 11 | 26027239 | A/C |
| 497 | 10 | 29442406 | G/C | 539 | 11 | 28064829 | G/A |
| 498 | 10 | 29442433 | C/T | 540 | 11 | 37290361 | C/G |
| 499 | 10 | 29442532 | C/T | 541 | 11 | 37291851 | G/A |
| 500 | 10 | 29443476 | C/T | 542 | 11 | 37391465 | G/C |
| 501 | 10 | 29443602 | G/A | 543 | 11 | 39843155 | T/A |
| 502 | 10 | 29446042 | G/A | 544 | 11 | 41124999 | C/T |
| 545 | 11 | 41125079 | G/A | 587 | 12 | 25114642 | C/A |
| 546 | 11 | 41125667 | C/T | 588 | 12 | 25115412 | C/T |
| 547 | 11 | 41126293 | T/C | 589 | 12 | 25119445 | G/A |
| 548 | 11 | 41126984 | G/A | 590 | 12 | 25119856 | T/A |
| 549 | 11 | 41127338 | C/T | 591 | 12 | 25120732 | C/A |
| 550 | 11 | 41128601 | G/A | 592 | 12 | 25123448 | T/C |
| 551 | 11 | 41128677 | T/C | 593 | 12 | 25123727 | A/G |
| 552 | 11 | 41128715 | G/A | 594 | 12 | 25126668 | T/G |
| 553 | 11 | 41297779 | C/T | 595 | 12 | 25127893 | G/C |
| 554 | 11 | 41550299 | A/G | 596 | 12 | 25129626 | C/T |
| 555 | 11 | 50612855 | C/A | 597 | 12 | 25130189 | G/A |
| 556 | 11 | 52983879 | C/T | 598 | 12 | 25135944 | A/G |
| 557 | 11 | 52984721 | T/C | 599 | 12 | 25136596 | T/C |
| 558 | 11 | 54799925 | C/T | 600 | 12 | 25137799 | A/G |
| 559 | 11 | 56305546 | G/A | 601 | 12 | 25138737 | A/G |
| 560 | 11 | 58361523 | C/T | 602 | 12 | 25139556 | G/A |
| 561 | 12 | 1310794 | A/G | 603 | 12 | 25141832 | T/C |
| 562 | 12 | 1311062 | A/G | 604 | 12 | 25144541 | T/C |
| 563 | 12 | 1313458 | G/A | 605 | 12 | 25146111 | G/A |
| 564 | 12 | 4859412 | A/G | 606 | 12 | 25149517 | C/A |
| 565 | 12 | 5630433 | T/C | 607 | 12 | 25152554 | T/A |
| 566 | 12 | 9010865 | A/C | 608 | 12 | 25154575 | C/T |
| 567 | 12 | 17006439 | C/T | 609 | 12 | 25155325 | T/C |
| 568 | 12 | 19175386 | C/A | 610 | 12 | 25159945 | T/G |
| 569 | 12 | 24624900 | C/T | 611 | 12 | 26146325 | T/C |
| 570 | 12 | 24624977 | C/G | 612 | 12 | 36252223 | G/A |
| 571 | 12 | 24625096 | A/G | 613 | 12 | 36253532 | G/A |
| 572 | 12 | 24625206 | T/C | 614 | 12 | 36253601 | G/A |
| 573 | 12 | 24625300 | A/G | 615 | 12 | 36254205 | G/A |
| 574 | 12 | 24625717 | G/A | 616 | 12 | 36254219 | T/C |
| 575 | 12 | 24625779 | T/C | 617 | 12 | 36257761 | G/A |
| 576 | 12 | 24626053 | A/C | 618 | 12 | 36258428 | G/A |
| 577 | 12 | 24626347 | G/A | 619 | 12 | 36286221 | T/C |
| 578 | 12 | 24626522 | C/T | 620 | 12 | 36292909 | G/A |
| 579 | 12 | 24626857 | G/A | 621 | 12 | 38746426 | C/T |
| 580 | 12 | 24626914 | G/A | 622 | 12 | 38751497 | G/A |
| 581 | 12 | 24626978 | T/C | 623 | 12 | 38780214 | G/A |
| 582 | 12 | 24627043 | C/G | 624 | 12 | 38789768 | C/T |
| 583 | 12 | 25112377 | G/A | 625 | 12 | 38789798 | C/T |
| 584 | 12 | 25113234 | C/A | 626 | 12 | 38789829 | G/A |
| 585 | 12 | 25113628 | C/T | 627 | 12 | 38797075 | G/T |
| 586 | 12 | 25114159 | G/A | 628 | 12 | 39476100 | C/T |
| 629 | 12 | 42442700 | C/T | 671 | 14 | 7905177 | C/T |
| 630 | 12 | 42616517 | T/G | 672 | 14 | 13948543 | G/A |
| 631 | 12 | 47361882 | G/A | 673 | 14 | 18682469 | A/T |
| 632 | 12 | 52369486 | C/T | 674 | 14 | 20060807 | C/T |
| 633 | 12 | 55696203 | C/A | 675 | 14 | 35362328 | C/T |
| 634 | 12 | 55865867 | C/G | 676 | 14 | 43393740 | G/T |
| 635 | 12 | 55918047 | C/A | 677 | 14 | 47778946 | A/G |
| 636 | 12 | 55921451 | G/C | 678 | 14 | 47780149 | T/C |
| 637 | 12 | 57276304 | T/G | 679 | 14 | 48476864 | A/G |
| 638 | 12 | 58033522 | T/C | 680 | 14 | 48625420 | A/G |
| 639 | 12 | 58513922 | T/A | 681 | 14 | 48820242 | G/A |
| 640 | 12 | 65627783 | G/A | 682 | 14 | 49745968 | T/C |
| 641 | 12 | 65659826 | T/A | 683 | 14 | 49746090 | C/A |
| 642 | 12 | 65664699 | A/G | 684 | 14 | 54292934 | T/C |
| 643 | 12 | 65667394 | A/G | 685 | 14 | 54293384 | A/G |
| 644 | 12 | 65675090 | C/T | 686 | 14 | 54295507 | C/T |
| 645 | 12 | 65677543 | A/G | 687 | 14 | 54295651 | T/C |
| 646 | 12 | 78576808 | T/C | 688 | 14 | 54295693 | A/G |
| 647 | 12 | 78597586 | G/C | 689 | 14 | 54296008 | C/A |
| 648 | 13 | 798029 | C/G | 690 | 14 | 54296036 | A/G |
| 649 | 13 | 1255836 | T/C | 691 | 14 | 57958208 | G/T |
| 650 | 13 | 2268617 | T/C | 692 | 14 | 58832278 | C/T |
| 651 | 13 | 15560337 | G/C | 693 | 14 | 61494669 | G/A |
| 652 | 13 | 20964453 | C/T | 694 | 14 | 61494679 | G/T |
| 653 | 13 | 24105520 | C/T | 686 | 14 | 54295507 | C/T |
| 654 | 13 | 24134649 | G/A | 687 | 14 | 54295651 | T/C |
| 655 | 13 | 24256053 | T/C | 688 | 14 | 54295693 | A/G |
| 656 | 13 | 25006383 | G/A | 689 | 14 | 54296008 | C/A |
| 657 | 13 | 28297902 | C/A | 690 | 14 | 54296036 | A/G |
| 658 | 13 | 30272517 | T/A | 691 | 14 | 57958208 | G/T |
| 659 | 13 | 30295418 | C/G | 692 | 14 | 58832278 | C/T |
| 660 | 13 | 38853749 | C/A | 693 | 14 | 61494669 | G/A |
| 661 | 13 | 58131558 | A/G | 694 | 14 | 61494679 | G/T |
| 662 | 13 | 60386901 | T/C | 695 | 15 | 854803 | C/T |
| 663 | 13 | 61807168 | T/C | 696 | 15 | 854865 | A/G |
| 664 | 13 | 61899795 | T/A | 697 | 15 | 855816 | G/C |
| 665 | 13 | 61964761 | C/G | 698 | 15 | 856407 | C/A |
| 666 | 13 | 66396102 | C/G | 699 | 15 | 856427 | T/C |
| 667 | 13 | 75500318 | A/C | 700 | 15 | 856524 | A/C |
| 668 | 13 | 76808776 | T/C | 701 | 15 | 3445985 | G/A |
| 669 | 13 | 80289907 | T/C | 702 | 15 | 3859136 | T/A |
| 670 | 14 | 1078888 | C/T | 703 | 15 | 3859139 | T/C |
| 704 | 15 | 3859161 | A/G | 746 | 15 | 21943725 | A/G |
| 705 | 15 | 3859270 | G/T | 747 | 15 | 21943773 | G/A |
| 706 | 15 | 3859400 | T/A | 748 | 15 | 21943863 | T/C |
| 707 | 15 | 3859530 | A/T | 749 | 15 | 21945029 | G/C |
| 708 | 15 | 3859596 | T/C | 750 | 15 | 21945083 | G/A |
| 709 | 15 | 3860698 | C/G | 751 | 15 | 21950914 | T/C |
| 710 | 15 | 3861238 | G/A | 752 | 15 | 21952976 | G/C |
| 711 | 15 | 3861243 | C/T | 753 | 15 | 21953019 | A/T |
| 712 | 15 | 3861271 | C/T | 754 | 15 | 21954250 | G/A |
| 713 | 15 | 3861303 | G/A | 755 | 15 | 21954265 | A/G |
| 714 | 15 | 3861409 | C/T | 756 | 15 | 21954340 | A/G |
| 715 | 15 | 3861423 | C/T | 757 | 15 | 21955296 | G/C |
| 716 | 15 | 3861551 | G/A | 758 | 15 | 21956732 | T/C |
| 717 | 15 | 3861670 | G/A | 759 | 15 | 21968749 | T/G |
| 718 | 15 | 3861700 | C/T | 760 | 15 | 21968764 | C/T |
| 719 | 15 | 3862023 | T/C | 761 | 15 | 21968776 | A/T |
| 720 | 15 | 3862047 | G/A | 762 | 15 | 21968790 | C/G |
| 721 | 15 | 3862106 | T/A | 763 | 15 | 21968803 | C/G |
| 722 | 15 | 3862447 | G/T | 764 | 15 | 21968821 | T/A |
| 723 | 15 | 3862453 | A/G | 765 | 15 | 21971053 | A/G |
| 724 | 15 | 3863143 | A/T | 766 | 15 | 21972758 | G/A |
| 725 | 15 | 3863172 | A/G | 767 | 15 | 22783795 | T/A |
| 726 | 15 | 3863520 | T/C | 768 | 15 | 23435084 | C/A |
| 727 | 15 | 3864434 | T/C | 769 | 15 | 25210561 | G/A |
| 728 | 15 | 3864891 | C/T | 770 | 15 | 31516790 | C/T |
| 729 | 15 | 3864977 | G/A | 771 | 15 | 46626270 | C/T |
| 730 | 15 | 3865100 | T/C | 772 | 15 | 47003773 | T/C |
| 731 | 15 | 6151460 | C/T | 773 | 15 | 47003774 | G/A |
| 732 | 15 | 7405786 | C/T | 774 | 15 | 47142978 | C/T |
| 733 | 15 | 7667085 | T/C | 775 | 15 | 47855286 | A/G |
| 734 | 15 | 11596539 | T/C | 776 | 15 | 50718950 | C/T |
| 735 | 15 | 18982765 | G/A | 777 | 15 | 51801557 | C/T |
| 736 | 15 | 21908192 | C/A | 778 | 15 | 51815844 | T/G |
| 737 | 15 | 21908219 | A/C | 779 | 15 | 51833383 | A/G |
| 738 | 15 | 21908226 | G/A | 780 | 15 | 51834989 | G/A |
| 739 | 15 | 21908259 | C/T | 781 | 15 | 68745093 | C/T |
| 740 | 15 | 21908277 | T/C | 782 | 15 | 72145778 | A/G |
| 741 | 15 | 21908285 | G/A | 783 | 15 | 72150928 | G/A |
| 742 | 15 | 21908294 | C/T | 784 | 15 | 73168535 | A/G |
| 743 | 15 | 21908301 | A/G | 785 | 15 | 73184314 | G/A |
| 744 | 15 | 21908325 | A/G | 786 | 15 | 73184854 | G/A |
| 745 | 15 | 21922633 | T/C | 787 | 15 | 73195323 | T/C |
| 788 | 15 | 78296283 | G/A | 830 | 17 | 66883407 | C/T |
| 789 | 15 | 78849104 | A/G | 831 | 17 | 68084654 | T/C |
| 790 | 16 | 2616304 | C/T | 832 | 17 | 68086716 | C/T |
| 791 | 16 | 14412544 | G/A | 833 | 17 | 68088059 | A/T |
| 792 | 16 | 16958033 | G/A | 834 | 17 | 68102675 | C/G |
| 793 | 16 | 23606490 | C/T | 835 | 17 | 68106116 | G/A |
| 794 | 16 | 23607353 | T/G | 836 | 17 | 68112940 | C/T |
| 795 | 16 | 24014404 | A/C | 837 | 17 | 69597308 | A/G |
| 796 | 16 | 48728588 | A/T | 838 | 17 | 70266858 | C/G |
| 797 | 16 | 56461568 | C/T | 839 | 17 | 71095391 | G/A |
| 798 | 17 | 1200887 | A/T | 840 | 18 | 998457 | A/G |
| 799 | 17 | 4860435 | C/T | 841 | 18 | 1060380 | T/C |
| 800 | 17 | 5893844 | T/C | 842 | 18 | 4522710 | G/A |
| 801 | 17 | 5893850 | A/G | 843 | 18 | 4522719 | C/G |
| 802 | 17 | 5894100 | G/A | 844 | 18 | 6949515 | G/A |
| 803 | 17 | 9012324 | A/G | 845 | 18 | 26340829 | G/T |
| 804 | 17 | 9811290 | C/A | 846 | 18 | 29037132 | G/A |
| 805 | 17 | 11740047 | C/T | 847 | 18 | 29037221 | C/G |
| 806 | 17 | 12333514 | A/G | 848 | 18 | 29039572 | C/T |
| 807 | 17 | 13767697 | C/T | 849 | 18 | 29852011 | A/T |
| 808 | 17 | 15997813 | G/C | 850 | 18 | 36755790 | G/A |
| 809 | 17 | 17696652 | T/A | 851 | 18 | 36792867 | C/G |
| 810 | 17 | 19043046 | T/C | 852 | 18 | 43468037 | G/A |
| 811 | 17 | 25539775 | A/G | 853 | 18 | 43468045 | C/T |
| 812 | 17 | 27810976 | G/T | 854 | 18 | 45692898 | C/T |
| 813 | 17 | 31769377 | T/C | 855 | 18 | 61261366 | A/G |
| 814 | 17 | 31769380 | A/G | 856 | 18 | 64294536 | A/G |
| 815 | 17 | 31769384 | A/C | 857 | 18 | 66361024 | A/T |
| 816 | 17 | 32480752 | G/A | 858 | 18 | 66448121 | A/G |
| 817 | 17 | 35606812 | T/C | 859 | 19 | 4474036 | C/T |
| 818 | 17 | 40410045 | C/T | 860 | 19 | 17686432 | C/G |
| 819 | 17 | 41573922 | A/G | 861 | 19 | 19096742 | G/A |
| 820 | 17 | 45404648 | T/C | 862 | 19 | 19116523 | C/A |
| 821 | 17 | 47901991 | G/A | 863 | 19 | 19118142 | C/G |
| 822 | 17 | 50488505 | A/G | 864 | 19 | 24132999 | C/T |
| 823 | 17 | 50582703 | A/C | 865 | 19 | 24218223 | T/C |
| 824 | 17 | 53320204 | T/C | 866 | 19 | 31657732 | C/T |
| 825 | 17 | 53452406 | C/T | 867 | 19 | 31671129 | G/A |
| 826 | 17 | 54731492 | G/A | 868 | 19 | 32756574 | C/T |
| 827 | 17 | 56249564 | C/T | 869 | 19 | 38281972 | G/A |
| 828 | 17 | 66866538 | T/G | 870 | 19 | 39433528 | G/A |
| 829 | 17 | 66866548 | G/A | 871 | 19 | 51772117 | T/C |
| 872 | 19 | 53139279 | G/A | 913 | 21 | 39563320 | G/A |
| 873 | 19 | 53140130 | C/T | 914 | 21 | 39563767 | G/A |
| 874 | 19 | 53141009 | A/G | 915 | 21 | 39564229 | A/G |
| 875 | 19 | 53141756 | T/C | 916 | 21 | 39661167 | A/C |
| 876 | 19 | 53142146 | A/C | 917 | 21 | 39663559 | C/T |
| 877 | 19 | 53142325 | C/T | 918 | 21 | 39667311 | A/G |
| 878 | 19 | 53142709 | C/A | 919 | 21 | 39674211 | C/A |
| 879 | 19 | 53142801 | T/G | 920 | 21 | 39681097 | A/G |
| 880 | 19 | 59799382 | A/C | 921 | 21 | 39681575 | C/T |
| 881 | 20 | 757936 | C/T | 922 | 21 | 39685141 | C/G |
| 882 | 20 | 2687568 | G/A | 923 | 21 | 39688171 | T/C |
| 883 | 20 | 3408392 | C/T | 924 | 21 | 39691339 | G/A |
| 884 | 20 | 4684821 | A/G | 925 | 21 | 39691901 | C/T |
| 885 | 20 | 25185724 | C/T | 926 | 21 | 39700447 | G/A |
| 886 | 20 | 25330788 | A/T | 927 | 21 | 39707519 | T/G |
| 887 | 20 | 26352362 | G/A | 928 | 21 | 39709105 | A/G |
| 888 | 20 | 26617208 | A/T | 929 | 21 | 39712741 | C/T |
| 889 | 20 | 26617271 | C/T | 930 | 21 | 39713282 | C/T |
| 890 | 20 | 26721144 | G/A | 931 | 21 | 39715270 | G/A |
| 891 | 20 | 26975740 | C/T | 932 | 21 | 41976803 | G/T |
| 892 | 20 | 27415669 | G/A | 933 | 21 | 41989155 | G/C |
| 893 | 20 | 28760749 | T/C | 934 | 21 | 42605942 | G/C |
| 894 | 20 | 37863763 | C/T | 935 | 21 | 42607179 | G/A |
| 895 | 20 | 38262044 | C/T | 936 | 21 | 42607683 | T/C |
| 896 | 20 | 44022390 | C/T | 937 | 21 | 42607981 | A/G |
| 897 | 20 | 44117660 | C/G | 938 | 21 | 42610805 | T/C |
| 898 | 20 | 44119346 | G/A | 939 | 21 | 42617857 | C/T |
| 899 | 20 | 44120218 | C/A | 940 | 21 | 42618822 | A/T |
| 900 | 20 | 50819827 | G/A | 941 | 21 | 42626055 | G/A |
| 901 | 21 | 4991242 | A/C | 942 | 21 | 42626364 | G/A |
| 902 | 21 | 17275168 | T/A | 943 | 21 | 42626685 | G/A |
| 903 | 21 | 31342331 | G/A | 944 | 21 | 42626874 | C/T |
| 904 | 21 | 33755040 | C/T | 945 | 21 | 42919557 | G/A |
| 905 | 21 | 39444648 | T/C | 946 | 21 | 46656298 | G/A |
| 906 | 21 | 39549848 | A/G | 947 | 22 | 2594738 | T/C |
| 907 | 21 | 39554987 | G/A | 948 | 22 | 6488828 | T/A |
| 908 | 21 | 39555337 | T/C | 949 | 22 | 7087618 | T/C |
| 909 | 21 | 39556971 | G/A | 950 | 22 | 11387209 | C/T |
| 910 | 21 | 39557117 | C/T | 951 | 22 | 11710763 | A/G |
| 911 | 21 | 39558448 | C/T | 952 | 22 | 20394968 | A/C |
| 912 | 21 | 39560480 | T/C | 953 | 22 | 20395012 | T/G |
| 954 | 22 | 20399268 | C/T | 984 | 24 | 40656018 | A/G |
| 955 | 22 | 23963118 | A/T | 985 | 24 | 40716102 | G/A |
| 956 | 22 | 37448335 | G/A | 986 | 25 | 787404 | A/C |
| 957 | 22 | 41592649 | G/A | 987 | 25 | 787528 | C/A |
| 958 | 22 | 43858480 | C/A | 988 | 25 | 4091535 | G/A |
| 959 | 22 | 43865478 | T/C | 989 | 25 | 13327307 | C/T |
| 960 | 22 | 43865511 | C/T | 990 | 25 | 13791395 | T/C |
| 961 | 22 | 46988971 | G/A | 991 | 25 | 20343310 | A/T |
| 962 | 22 | 47002481 | A/C | 992 | 25 | 26620938 | A/G |
| 963 | 22 | 48817370 | C/T | 993 | 25 | 29093403 | A/T |
| 964 | 22 | 50203275 | G/A | 994 | 25 | 29619051 | T/C |
| 965 | 23 | 76654 | A/G | 995 | 25 | 30628106 | C/G |
| 966 | 23 | 4588565 | A/G | 996 | 25 | 32701433 | C/T |
| 967 | 23 | 21427546 | A/G | 997 | 25 | 37388238 | C/T |
| 968 | 23 | 30978805 | C/G | 998 | 25 | 39719108 | C/T |
| 969 | 23 | 33523412 | A/G | 999 | 26 | 3412783 | T/C |
| 970 | 23 | 49558064 | G/A | 1000 | 26 | 5647776 | T/C |
| 971 | 23 | 53179718 | G/A | 1001 | 26 | 7902723 | G/A |
| 972 | 24 | 8839062 | C/G | 1002 | 26 | 15098976 | T/C |
| 973 | 24 | 8839074 | C/T | 1003 | 26 | 15099204 | G/T |
| 974 | 24 | 8872749 | G/A | 1004 | 26 | 15099391 | C/T |
| 975 | 24 | 10555981 | G/A | 1005 | 26 | 15099485 | C/T |
| 976 | 24 | 20264728 | C/A | 1006 | 26 | 15099491 | G/A |
| 977 | 24 | 21423158 | C/T | 1007 | 26 | 15680228 | A/G |
| 978 | 24 | 28968472 | T/C | 1008 | 26 | 18221835 | G/A |
| 979 | 24 | 28988429 | T/C | 1009 | 26 | 18221987 | C/T |
| 980 | 24 | 29007770 | G/A | 1010 | 26 | 28701229 | G/A |
| 981 | 24 | 29012050 | C/A | 1011 | 26 | 43050080 | C/T |
| 982 | 24 | 29018060 | G/T | 1012 | 26 | 43709369 | G/A |
| 983 | 24 | 30294197 | G/T | —— | | | |
